# Supplementary material for: Minimizing the Anticodon-Recognized Loop of Methanococcus jannaschii Tyrosyl-tRNA Synthetase to Improve the Efficiency of Incorporating Noncanonical Amino Acids
Source: Biomolecules. 2023 Mar 28;13(4):610. doi: 10.3390/biom13040610 (PMC10135938; doi:10.3390/biom13040610)
Supplement: Supplementary file 1 [file biomolecules-13-00610-s001.zip › Supplementary/Supporting Information final.pdf]

Minimizing the Anticodon-Recognized Loop of *Methanococcus jannaschii* Tyrosyl-tRNA Synthetase to Improve the Efficiency of Incorporating Noncanonical Amino Acids

Zhiyang Hu †, Jinming Liang †, Taogeng Su, Di Zhang, Hao Li, Xiangdong Gao *, Wenbin Yao * and Xiaoda Song *

Table S1. Primers used for this study.

Sequence	Usage
*Mj*Loop-1-s: accctgtgcggcTTGACAGTTAATAGCTATGAGGAG<br>*Mj*Loop-1-as: caagccgcacagGGTTAAAGGATATTCAAGGAAGT	*Mj*TyrRS-Loop1
*Mj*Loop-2-s: accggaagtggtTTGACAGTTAATAGCTATGAGGAG<br>*Mj*Loop-2-as: caaaccacttccGGTTAAAGGATATTCAAGGAAGT	*Mj*TyrRS-Loop2
*Mj*Loop-3-s: accggaggtagtggtTTGACAGTTAATAGCTATGAGGAG<br>*Mj*Loop-3-as: caaaccactacctccGGTTAAAGGATATTCAAGGAAGT	*Mj*TyrRS-Loop3
eGFP150-TGA-s: CAGCCACtgaGTGTACATCATGGCGGACAAGC<br>eGFP150-TGA-as: TGTACACtcaGTGGCTGTTGTAGTTATACTCCAGCT	eGFP150-TGA
*Mj*tRNA-TGA-s: CGGCGGACTtcaAATCCGCATGGCAGGGGTTCAAATC<br>*Mj*tRNA-TGA-as: GGATTtgaAGTCCGCCGTTCTGCCCTGCTGAACT	*Mj*tRNA-TGA
eGFP150-TAA-s:CAGCCACtaaGTGTACATCATGGCGGACAAGC<br>eGFP150-TAA-as:TGTACACttaGTGGCTGTTGTAGTTATACTCCAGCT	eGFP150-TAA
*Mj*tRNA-TAA-s: CGGCGGACTttaAATCCGCATGGCAGGGGTTCAAATC<br>*Mj*tRNA-TAA-as: GGATTtaaAGTCCGCCGTTCTGCCCTGCTGAACT	MjtRNA-TAA
eGFP150-TAGA-s:CAGCCACtagaGTGTACATCATGGCGGACAAGC<br>eGFP150-TAGA-as:TGTACACtctaGTGGCTGTTGTAGTTATACTCCAGCT	eGFP150-TAGA
*Mj*tRNA-TAGA-s: CGGCGGACTtctaAATCCGCATGGCAGGGGTTCAAATC<br>*Mj*tRNA-TAGA-as: GGATTtagaAGTCCGCCGTTCTGCCCTGCTGAACT	*Mj*tRNA-TAGA
eGFP150-TAGT-s:CAGCCACtagtGTGTACATCATGGCGGACAAGC<br>eGFP150-TAGT-as:TGTACACactaGTGGCTGTTGTAGTTATACTCCAGCT	eGFP150-TAGT
*Mj*tRNA-TAGT-s: CGGCGGACTactaAATCCGCATGGCAGGGGTTCAAATC<br>*Mj*tRNA-TAGT-as: GGATTtagtAGTCCGCCGTTCTGCCCTGCTGAACT	*Mj*tRNA-TAGT
eGFP150-TAGC-s:CAGCCACtagcGTGTACATCATGGCGGACAAGC<br>eGFP150-TAGC-as:TGTACACgctaGTGGCTGTTGTAGTTATACTCCAGCT	eGFP150-TAGC
*Mj*tRNA-TAGC-s: CGGCGGACTgctaAATCCGCATGGCAGGGGTTCAAATC<br>*Mj*tRNA-TAGC-as: GGATTtagcAGTCCGCCGTTCTGCCCTGCTGAACT	*Mj*tRNA-TAGC
eGFP150-TAGG-s:CAGCCACtaggGTGTACATCATGGCGGACAAGC<br>eGFP150-TAGG-as:TGTACACcctaGTGGCTGTTGTAGTTATACTCCAGCT	eGFP150-TAGG
*Mj*tRNA-TAGG-s: CGGCGGACTcctaAATCCGCATGGCAGGGGTTCAAATC<br>*Mj*tRNA-TAGG-as: GGATTtaggAGTCCGCCGTTCTGCCCTGCTGAACT	*Mj*tRNA-TAGG
eGFP150-TGAA-s:CAGCCACtgaaGTGTACATCATGGCGGACAAGC<br>eGFP150-TGAA-as:TGTACACttcaGTGGCTGTTGTAGTTATACTCCAGCT	eGFP150-TGAA
*Mj*tRNA-TGAA-s: CGGCGGACTttcaAATCCGCATGGCAGGGGTTCAAATC<br>*Mj*tRNA-TGAA-as: GGATTtgaaAGTCCGCCGTTCTGCCCTGCTGAACT	*Mj*tRNA-TGAA
eGFP150-TGAT-s:CAGCCACtgatGTGTACATCATGGCGGACAAGC<br>eGFP150-TGAT-as:TGTACACatcaGTGGCTGTTGTAGTTATACTCCAGCT	eGFP150-TGAT
*Mj*tRNA-TGAT-s: CGGCGGACTatcaAATCCGCATGGCAGGGGTTCAAATC<br>*Mj*tRNA-TGAT-as: GGATTtgatAGTCCGCCGTTCTGCCCTGCTGAACT	*Mj*tRNA-TGAT
eGFP150-TGAC-s:CAGCCACtgacGTGTACATCATGGCGGACAAGC<br>eGFP150-TGAC-as:TGTACACgtcaGTGGCTGTTGTAGTTATACTCCAGCT	eGFP150-TGAC
*Mj*tRNA-TGAC-s:CCGGCGGACTgtcaAATCCGCATGGCAGGGGTTCAAATC<br>*Mj*tRNA-TGAC-as: GGATTtgacAGTCCGCCGTTCTGCCCTGCTGAACT	*Mj*tRNA-TGAC
eGFP150-TGAG-s:CAGCCACtgagGTGTACATCATGGCGGACAAGC<br>eGFP150-TGAG-as:TGTACACctcaGTGGCTGTTGTAGTTATACTCCAGCT	eGFP150-TGAG
*Mj*tRNA-TGAG-s: CGGCGGACTctcaAATCCGCATGGCAGGGGTTCAAATC<br>*Mj*tRNA-TGAG-as: GGATTtgagAGTCCGCCGTTCTGCCCTGCTGAACT	*Mj*tRNA-TGAG
eGFP150-TAAA-s:CAGCCACtaaaGTGTACATCATGGCGGACAAGC<br>eGFP150-TAAA-as:TGTACACtttaGTGGCTGTTGTAGTTATACTCCAGCT	eGFP150-TAAA

*Mj*tRNA-TAAA-s: CGGCGGACTtttaAATCCGCATGGCAGGGGTTCAAATC<br>*Mj*tRNA-TAAA-as: GGATTtaaaAGTCCGCCGTTCTGCCCTGCTGAACT	*Mj*tRNA-TAAA
eGFP150-TAAT-s:CAGCCACtaatGTGTACATCATGGCGGACAAGC<br>eGFP150-TAAT-as:TGTACACattaGTGGCTGTTGTAGTTATACTCCAGCT	eGFP150-TAAT
*Mj*tRNA-TAAT-s: CGGCGGACTattaAATCCGCATGGCAGGGGTTCAAATC<br>*Mj*tRNA-TAAT-as: GGATTtaatAGTCCGCCGTTCTGCCCTGCTGAACT	*Mj*tRNA-TAAT
eGFP150-TAAC-s:CAGCCACtaacGTGTACATCATGGCGGACAAGC<br>eGFP150-TAAC-as:TGTACACgttaGTGGCTGTTGTAGTTATACTCCAGCT	eGFP150-TAAC
*Mj*tRNA-TAAC-s: CGGCGGACTgttaAATCCGCATGGCAGGGGTTCAAATC<br>*Mj*tRNA-TAAC-as: GGATTtaacAGTCCGCCGTTCTGCCCTGCTGAACT	*Mj*tRNA-TAAC
eGFP150-TAAG-s:CAGCCACtaagGTGTACATCATGGCGGACAAGC<br>eGFP150-TAAG-as:TGTACACcttaGTGGCTGTTGTAGTTATACTCCAGCT	eGFP150-TAAG
*Mj*tRNA-TAAG-s: CGGCGGACTcttaAATCCGCATGGCAGGGGTTCAAATC<br>*Mj*tRNA-TAAG-as: GGATTtaagAGTCCGCCGTTCTGCCCTGCTGAACT	*Mj*tRNA-TAAG

Table S2. The MjTyrRS mutants used in this study.

Substrate/position	32	65	70	107	108	109	158	159	162	167	286
MjTyrRS(WT)	Y	L	H	E	F	Q	D	I	L	A	D
O-Me-Tyr	Q	L	H	T	F	Q	A	I	P	A	D
4-N_3-Phe	T	L	H	N	F	Q	P	L	Q	A	D
4-I-Phe	L	L	H	S	F	Q	P	L	E	A	D
4-$B(OH)_2$-Phe	S	A	M	E	F	Q	S	I	E	A	D
3-NO_2-Tyr	H	L	C	E	F	Q	S	A	R	A	D
4-NO_2-Phe	L	V	H	E	W	M	G	A	L	A	D
AcFRA.1	L	L	H	E	F	Q	G	C	R	D	R
AzFRA.1	L	L	H	E	F	Q	V	M	D	Y	R
AzFRA.2	L	L	H	T	Y	M	G	C	R	A	R

Supplementary Sequence 1 The sequence of TyrRSs from giant viruses

Acanthamoeba castellanii mamavirus

MENTDYTNNEHRLNQLLSIAEECETLDRLKQLVDSGRIFTAYNGFEPSGRIHIAQALITVMNTNKIIECGGQMIIYIAD

WFAKMNLKMNGDINKIRELGRYFIEVFKACGINLDGTRFIWASEFIASNPSYIERMLDIAEFSTISRVKRCCQIMGRNE

SDCLKASQIFYPCMQAADVFELVPEGIDICQLGIDQRKVNMLAIEYANDRGLKIPISLSHHMLMSLSGPKKKMSKSD

PQGAIFMDDTEQEVSEKISRAYCTDEIFDNPIFEYIKYLLLRWFGTLNLCGKIYTDIESIQEDFSSMNKRELKTDVANYI

NTIIDLVREHFKKPELSELLSNVKSYQQPSK

Acanthamoeba castellanii mimivirus

MENTDHTNNEHRLTQLLSIAEECETLDRLKQLVDSGRIFTAYNGFEPSGRIHIAQALITVMNTNNIIECGGQMIIYIAD

WFAKMNLKMNGDINKIRELGRYFIEVFKACGINLDGTRFIWASEFIASNPSYIERMLDIAEFSTISRVKRCCQIMGRNE

SDCLKASQIFYPCMQAADVFELVPEGIDICQLGIDQRKVNMLAIEYANDRGLKIPISLSHHMLMSLSGPKKKMSKSD

PQGAIFMDDTEQEVSEKISRAYCTDETFDNPIFEYIKYLLLRWFGTLNLCGKIYTDIESIQEDFSSMNKRELKTDVANYI

NTIIDLVREHFKKPELSELLSNVKSYQQPSK

Acanthamoeba polyphaga lentillevirus

MENTDYTNNEHRLNQLLSIAEECETLDRLKQLVDSGRIFTAYNGFEPSGRIHIAQALITVMNTNKIIECGGQMIIYIAD

WFAKMNLKMNGDINKIRELGRYFIEVFKACGINLDGTRFIWASEFIASNPSYIERMLDIAEFSTISRVKRCCQIMGRNE

SDCLKASQIFYPCMQAADVFELVPEGIDICQLGIDQRKVNMLAIEYANDRGLKIPISLSHHMLMSLSGTKKKMSKSD
PQGAIFMDDTEQEVSEKISRAYCTDEIFDNPIFEYIKYLLLRWFGTLNLCGKIYTDIESIQEDFSSMNKRELKTDVANYI
NTIIDLVREHFKKPELSELLSNVKSYQQPSK

Acanthamoeba polyphaga mimivirus

MENTDHTNNEHRLTQLLSIAEECETLDRLKQLVDSGRIFTAYNGFEPSGRIHIAQALITVMNTNNIIECGGQMIIYIAD
WFAKMNLKMNGDINKIRELGRYFIEVFKACGINLDGTRFIWASEFIASNPSYIERMLDIAEFSTISRVKRCCQIMGRNE
SDCLKASQIFYPCMQAADVFELVPEGIDICQLGIDQRKVNMLAIEYANDRGLKIPISLSHHMLMSLSGPKKKMSKSD
PQGAIFMDDTEQEVSEKISRAYCTDETFDNPIFEYIKYLLLRWFGTLNLCGKIYTDIESIQEDFSSMNKRELKTDVANYI
NTIIDLVREHFKKPELSELLSNVKSYQQPSK

Acanthamoeba polyphaga moumouvirus

MEQQILSTEEKITRLISICEECETPDRLEELIKSGKKFTAYNGFEPSGRIHIAQALITVMNTNEIISCGGRMIIYIADLFAK
MNKKMDSDLDKIKDVGLYFIEVFKACGINMSETKFIWSSDLFNKNPEKYFTRLLDISEETSLSRSKRCCQIMGRKDGD
ELSVSQIIYPCMQVADIFELEPEGIDICQLGVDQRKVNMLALEYAKKNGLKVPVILSHHMLMGLGGPKNKMSKSDP
KSAIFMEDSYEEIKEKILRAFCTDDINENPIFEYIKHILFRWYGKLELCGKNYDNMSDIEKDFCDMNKRELKNNVAEY
INKIIEPVRIHFQQPHLKNLLDKVSSYRVTK

Cafeteria roenbergensis virus BV-PW1

MNQFKELLTNVENISLIYNDSDNRVMFTMKSHLLYFLTRFKNSDIVKIIEKDNLKYFMLDEISSDNYLDIYDKFIKEYQ
IYDLDYDNFIDQRVQNLLSISEECDTIDRLKELVESFKNFTAYNGFEPSGRIHIAQALITVMNTNTIIQNGGSMIIYIAD
WFAQMNHKMGGDLEKIRDVGKYFIEVFKACGINMSGTKFIWASEFFNDNATKYFERMLHIANQTSLARAKRCCQI
MGRREGDELSSSQIIYPCMQVADIFELNPNGIDICQLGVDQRKVNMLAIEYAKRNNLKTPIILSHHMLMGLKGSKN
KMSKSDPENAIFMEDSREDIFKKIHKAYCTDEIIDNPIYEYIKYILLRWFGTITLCGQKYNNIDDINKNFATMLKKDLK
NDVADYIDYILEPVRRHFQDPQMAELYSRVRSYTN

Catovirus CTV1

MNIQENIKTIKSIGEEIIGGDSLERLLTHKQRVYAYDGFEPSGRMHIAQGLLRTHNVNKFIDSGVHFKFWVADWFAL
MNLKLGGDIKKIQQAGKDMILMWKACDMNLDKVDKDGNKMIEFLWSSEEINKRPDEYWKLVLDIATKFSLNRIK
KCTQIMGRKEEDEIFNTILDLNDKIVDLFNNCDFEQKKKDDILGLLKKLLDTKLDELAASQIFYPVMQCADVFFLGI
DIASLGMDQRKVNTLALEYCDKIKRKNKPIIISHHMLKGLDGSDKMSKSNPDNTIFMDDSETEIIRKIKKSFCEPGNI
DKNPLLDWIEHLILPIKKTIVIKTKNDIGEEIDMSFDNANLIADKFKNGYIHPKCLKNAVTTVLVNTLIPIQQKYIALK
KNDN

Cotonvirus japonicus

MDNFTRFCAELSQNRDINVINISENEIIFEFVDKSLYFTNKFEEDRIIDITFIDNQKYLCVTREDVDSNFIPDSTYVIKCH
DSYVETFDVDVKKRVDQLVAISEECDDRDRLRDLIISGKKFVAYNGFEPSGRIHIAQAVITVLNTNTIIENGGRMIIYIA
DWFAQLNHKLGGDLEKIQNVGRYFIEVFKACGIDTSNTEFIWASEFFAKNPSRYFQRMMDVSINATLARTKRCCQI
MGRAEGDNLSSSQIIYPCMQTADIFELVEGGVDICQLGVDQRKVNMFALDYANSQKLKLPVILSHHMIMGLRGPKH
KMSKSDPLNAIFMEDTTQEIHDKISKAFCPDTHLDNPIFEYIKYILLRWFGNLTLCDNFYTNIDFIIQDFPHFDKKRLK
TDVANYIDRIIKPVRDHFAQDELKDLYDKVIN

Edafosvirus sp.

MSEDKYNIITRNLQEIIGSDDLKELLKNKNPKIYWGTAPTGRIHIAYFLPLLKIADFLDAGCHVKILIADLHAYLDNM
KSSLDQLNSRCEYYTHMIKEILKVLNVNLSKLEFVKGTDFQLNKEYTMDVYKAHNMISYNEARHAGAEVVKQTDN
PNINSLLYPTLQALDEQYLDVDIQFGGNDQRKIFMHARKILPSIGYKKRIYLMNSIIPALSKVSLKDPQQESTITNKKM
SSSDNTSKIDVLEDEKSIKKKINSTYCLEGDISDNTLLTMSKLLIFPILNRLNQKLIIQRPEKYGGNIIYDNYTLLESDFET
KKLHPSDLKNGISLFIINLIKPIQIEFQKEELVKLLQKAYS

Fadolivirus 1

MELTVDQKYELITRNLQEKIVDEEIMKKILAARPLKIYWGTAPTSLCHLGYFAPMFKIIDFLQAGCEVTILIADLHAVL

DNMKSTFEQVDARTKVYTVLIQELLKSLNVDISRLKFVKGTDYQLSKEYTLDMYKAHTLISVSEAKHAGAEVVKQS
DNPKMTGLLYPTLQALDEQYLGSDAELGGIDQRKIFVHARAIMPALGYKKRFHLMNRMVPGLRFEKTTPLQSVQN
EQKESLKEKVLSLVNSEADEKVLADRLQSLLENNDQSNIQLEKMSSSNADSKISLLDTRNQIRSKINKAYCLPGDVD
DNCLLTMLDKIVFPVLKLKGLDFVINRKEEHGGKLVYTNIDDVTNDFKTEKLHPADLKLGMVDSLDLMMEPLRNA
FKSKESVQLLNKAYPQK

Harvfovirus sp.

MQSRYDSITAGVKEVINAKYLKALLEDASYSPKGYWGTAPTGRIHIGYLRAMFVIADLVNANCEVIILLADIHAFLDS
RKSPQDNQLRTQYYQIMITELLKLLNVDMSKIKFVIGSSFQETKEYMRELLAIANVTNCSNAKDAGTEVVMQDSDP
KLTSLLYPIMQVLDEKYLNADFELGGVDQRKIFTFGIDHVHRHTKHKITYLMNSMVPALSGISGDSGVKMSASGSVG
KLDLLDSPKELLKKIKKVYCLQGDIADNTLLQLCKHIVFPLLSRVNKIMIVTKKFDHVESEIKNYEDLENLFASGLHP
DDFKTSVALTIGELLKPIRDKFLETDMVTLLKKAYDSK

Hirudovirus strain Sangsue

MENTDYTNNEHRLNQLLSIAEECETLDRLKQLVDSGRIFTAYNGFEPSGRIHIAQALITVMNTNKIIECGGQMIIYIAD
WFAKMNLKMNGDINKIRELGRYFIEVFKACGINLDGTRFIWASEFIASNPSYIERMLDIAEFSTISRVKRCCQIMGRNE
SDCLKASQIFYPCMQAADVFELVPEGIDICQLGIDQRKVNMLAIEYANDRGLKIPISLSHHMLMSLSGPKKKMSKSD
PQGAIFMDDTEQEVSEKISRAYCTDEIFDNPIFEYIKYLLLRWFGTLNLCGKIYTDIESIQEDFSSMNKRELKTDVANYI
NTIIDLVREHFKKPELSELLSNVKSYQQPSK

Hokovirus HKV1

MNKLTIDEKRELITKNLQEILGKQELDNLLLQEEIKIYWGTAPTGRIHIGYFIQFLKIVDYIKAGCKVKILIADLHAVLD
NLKTSMELLNARTEYYITMIKEVLLSLNVDLDLIEFVKGSDYQLSQNYTLDVYKLNSKISYQEAKHAGAEVVKQTQ
NPTMTGLLYPSLQALDEHYLDVHIQSGGIDQRKIFTFARSNLNLLGYKKSIHLMTPMVQGLRFNKKEIKNVITKEEK
LKLLNIDFDLPDNYVDTLYKKYINNDTIKDDKMSSSNNDSKIDLLDTKNEIKKKINKSYCLPCDVEDNCLMDLLEKV
IFPVLSYKNQQFIINRKEKFGGIIIYDSFDNIKNDFALGKLHPQDFKLGITDSLNYILEPIRNSFETKERKLLLKKAYN

Homavirus sp.

MTNLSVDEKYNLITRNLQEVLGSEADIKSILAERPLNIYWGTAATGKIHIGYIFQMMKIADYLQAGCNVTILIADLHA
YLDNMKSSLELINYRTEYYTKMIQTILITMGVDISRLTFVKGTSFQLSEKYTMDMYRANSMISVKDAQHAGAEVVKQ
TDNPIMNGLLYPTLQALDEQYLNIDAETSGIDQRKLFAHAKMIMPKLGYKKRNYFMTEMISGLRFVKKQEQSHSQP
VKLNRDDIINLLNIESDDALINNLQEMIDKYNKEKELKNNIQLEKMSSSDNNSKIDLLDTKNQIKAKINKCYCLAGD
IKDNCLMNILEKLLFPMMKYKGLDFIINRPDKYGGPIVYTEFDNVKNDFEKELLHPSDFKMGITDGFDTIIKPIRDIFG
TPELQKLIKQAYP

Klosneuvirus KNV1

MELSVDEKFNLITRNLQETILDEVIMKKIIAARPLKVYWGTAPTSAPSVAYFVPMMKIRDLVMAGCEVTILIADLHAI
LDNLKSTFEQVGHRSEYYITMIKALLTSLNIDVNKIKFVKGTEFQLSKEYTLDMYKAHSLITVNEAKHAGAEVVKQA
EHPKMTGLMYPTLQALDEQYLGADVEIGGIDQRKLFMHARNIMPQLGYKKRMYLMNKMISGLRTQKQEPKEGEN
IQDQKMSSSNQDSKIDLLDSKNQLKAKINKAYCFPGDINDNCLLEMLENLVFPILHIKGLNFVIERKEEYGGNITYN
NIDNVKNDFKSEKLHPMDLKQGMINNLDLILDSIRKTFETPEMKKLIKMAYS

Marseillevirus LCMAC201

MSNFDLIVKGLHEIVGKDLLEKIAVERVVKVYWGTAPTGRIHIGYYIPLLKIAELVKAGCEVYILIADLHAMLDNLKS
TEKQVHNRSDYYTRTIKNMLTQLNVDLDKVTFILGSTYQTKPEYTMDMYRLNMLCKVSDARHAGSEVVKQSNNP
MMTGLLYPSLQALDEEYLHVDAQLGGIDQRKIFMFAREFLPKIGYRKRIHLMTPMVPGIRHCASSKSMDSQEKMSA
SDTKTKLDMLDTRNQIKKKIASAFCIPGDVTDNSILDILERVLFPLLKHLSKDFVIIRREEHGGPITFTNFNKVREAFVA
EKLHPADLKAGIVHTLDIFIEPVRKEFESREWRQVLKHSYAED

Marseillevirus LCMAC202

MSNYDLIVKDLHEIVGEDILKKIAAERTVKVYWGTAPTGRIHIGYYTPLLKIAELVQAGCEVTILIADLHAMLDNLKS

TEKQVHNRSDYYTRIIKNMLTQLNVDLDKVTFVRGSSFQFTPEYTMDMYRLNTVCSFHDAQHAGAEVVKQTSNPI
MTGLLYPSLQALDEEYLHVDAQLGGIDQRKIFMFAREFLPKIGYRKRVHLLTPMVPGLRCCASTQDNEDKMSTSNT
KTKLDMLDSRNEIKKKIAAAYCLPGDITDNSVLDILDRVLFPLLRHLSKDFIIFRREEHGGPIIFTNFDQVREAFSTQQL
HPADLKAGT

Megavirus chiliensis

MNQFKELLTNVENISLIYNDSDNRVMFTMKSHLLYFLTRFKNSDIVKIIEKDNLKYFMLDEISSDNYLDIYDKFIKEYQ
IYDLDYDNFIDQRVQNLLSISEECDTIDRLKELVESFKNFTAYNGFEPSGRIHIAQALITVMNTNTIIQNGGSMIIYIAD
WFAQMNHKMGGDLEKIRDVGKYFIEVFKACGINMSGTKFIWASEFFNDNATKYFERMLHIANQTSLARAKRCCQI
MGRREGDELSSSQIIYPCMQVADIFELNPNGIDICQLGVDQRKVNMLAIEYAKRNNLKTPIILSHHMLMGLKGSKN
KMSKSDPENAIFMEDSREDIFKKIHKAYCTDEIIDNPIYEYIKYILLRWFGTITLCGQKYNNIDDINKNFATMLKKDLK
NDVADYIDYILEPVRRHFQDPQMAELYSRVRSYTN

Megavirus courdo7-1

MEQQILSTEEKITRLISICEECETPDRLEELIKSGKKFTAYNGFEPSGRIHIAQALITVMNTNEIISCGGRMIIYIADLFAK
MNKKMDSDLDKIKDVGLYFIEVFKACGINMSETKFIWSSDLFNKNPEKYFTRLLDISEETSLSRSKRCCQIMGRKDGD
ELSVSQIIYPCMQVADIFELEPEGIDICQLGVDQRKVNMLALEYAKKNGLKVPVILSHHMLMGLGGPKNKMSKSDP
KSAIFMEDSYEEIKEKILRAFCTDDINENPIFEYIKHILFRWYGKLELCGKNYDNMSDIEKDFCDMNKRELKNNVAEY
INKIIEPVRIHFQQPHLKNLLDKVSSYRVTK

Megavirus courdo7-2

MNQFKELLTNVENISLIYNDSDNHVMFTMKSHLSYFLTRFKNSDVVKIIEKDNLKYFMLDEIASDNYLDVYDKFMK
EYQIYDLDYDNFIDQRVQNLLSISEECDTIDRLKELVESFKNFTAYNGFEPSGRIHIAQALITVMNTNTIIQNGGSMIIYI
ADWFAQMNHKMGGDLEKIRDVGKYFIEVFKACGINMSGTKFIWASEFFNDNATKYFERMLHIANQTSLARAKRC
CQIMGRREGDELSSSQIIYPCMQVADIFELNPNGIDICQLGVDQRKVNMLAIEYAKRNNLKTPIILSHHMLMGLKGS
KNKMSKSDPENAIFMEDSREDIFKKIYKAYCTDEIIDNPIYEYIKYILLRWFGTITLCGQKYNNIDDINKNFATMQKKD
LKNDVADYIDYILEPVRRHFQDPQMAELYSRVRSYTN

Megavirus courdo11

MNQFKELLTNVENISLIYNDSDNHVMFTMKSHLSYFLTRFKNSDVVKIIEKDNLKYFMLDEISSNNYLDIYDKFMKE
YQIYDLDYDNFIDQRVQNLLSISEECDTIDRLKELVESFKNFTAYNGFEPSGRIHIAQALITV
MNTNTIIQNGGSMIIYIADWFAQMNHKMGGDLEKIKDVGKYFIEVFKACGINMSGTKFIWASEFFNDNATKYFERM
LHIANKTSLARAKRCCQIMGRREGDELSSSQIIYPCMQVADIFELNPNGIDICQLGVDQRKVNMLAIEYAKRNNLKT
PIILSHHMLMGLKGSKNKMSKSDPENAIFMEDSREDIFKKIHKAYCTDEIIDNPIYEYIKYILLRWFGTITLCGQKYNNI
DDINKNFATMLKKDLKNDVADYIDYILEPVRRHFQDPQMAELYSRVRSYTN

Megavirus lba isolate LBA111

MNQFKELLTNVENISLIYNDSDNHVMFTMKSHLSYFLTRFKNSDVVKIIEKDNLKYFMLDEISSDNYLDIYDKFIKEY
QIYDLDYDNFIDQRVQNLLSISEECDTIDRLKELVESFKNFTAYNGFEPSGRIHIAQALITVMNTNTIIQNGGSMIIYIA
DWFAQMNHKMGGDLEKIKDVGKYFIEVFKACGINMSGTKFIWASEFFNDNATKYFERMLHIANQTSLARAKRCC
QIMGRREGDELSSSQIIYPCMQVADIFELNPNGIDICQLGVDQRKVNMLAIEYAKRNNLKTPIILSHHMLMGLKGSK
NKMSKSDPENAIFMEDSREDIFKKIYKAYCTDEIVDNPIYEYIKYILLRWFGTITLCGQKYNNIDDINKNFATMQKKD
LKNDVADYIDYILEPVRRHFQDPQMAELYSRVRSYTN

Megavirus vitis

MNQFKELLTNVENISLIYNDSDNHVMFTMKSHLSYFLTRFKNSDVVKIIEKDNLKYFMLDEIASDNYLDVYDKFIKE
YHIYDLDYDNFIDQRVQNLLSISEECDTIDRLKELVESFKNFTAYNGFEPSGRIHIAQALITVMNTNTIIQNGGSMIIYI
ADWFAQMNHKMGGDLEKIRDVGKYFIEVFKACGINMSGTKFIWASEFFNDNATKYFERMLHIANQTSLARAKRC
CQIMGRREGDELSSSQIIYPCMQVADIFELNPNGIDICQLGVDQRKVNMLAIEYAKRNNLKTPIILSHHMLMGLKGS
KNKMSKSDPENAIFMEDSREDIFKKIYKAYCTDEIIDNPIYEYIKYILLRWFGTITLCGQKYNNIDDINKNFATMQKKD

LKNDVADYIDYILEPVRRHFQDPQMAELYSRVRSYTN

Mimivirus Bombay

MENTDHTNNEHRLTQLLSIAEECETLDRLKQLVDSGRIFTAYNGFEPSGRIHIAQALITVMNTNNIIECGGQMIIYIAD
WFAKMNLKMNGDINKIRELGRYFIEVFKACGINLDGTRFIWASEFIASNPSYIERMLDIAEFSTISRVKRCCQIMGRNE
SDCLKASQIFYPCMQAADVFELVPEGIDICQLGIDQRKVNMLAIEYANDRGLKIPISLSHHMLMSLSGPKKKMSKSD
PQGAIFMDDTEQEVSEKISRAYCTDETFDNPIFEYIKYLLLRWFGTLNLCGKIYTDIESIQEDFSSMNKRELKTDVANYI
NTIIDLVREHFKKPELSELLSNVKSYQQPSK

Mimivirus LCMiAC01

MTELTTFDFITRNLEPLVGNDAVRLLEIIKDRPLKLYWGTAPTGAPHFGYLVPLRKIADFLKAGCEVTIMFADIHAML
DNLKTKPDLIQIRTTCYEMIIKRILQIFKVPLHKLRFIRGSEFQTSPEYINKFYELTAYTTIKQAIHAGAEVVKQTSSPKLS
SVIYPLLQALDEEFLGVDGQFGGIDQAKIFMFAKDQLPRIGISKRIHLMNALIPGLKKGGKMSSSIPYSKIDFRDTDEDI
NRKILGAFSIDGHVGNGKNKNCMLAMMEHVIYPTLNGESFMIKRDRLYGGNIKITSYKELEKMFQLNKLSSIDLKIS
MVPYIQKLIKPIRAMISEQSELFDSACWHAHHANVPIISSSLQSIQSTQLKKMTKKMTKKITKKTIELSLEDKIACELK
AIQKMEQHIIFKKKKVEELRRQMGEKE

Mimivirus reunion

MENTDYTNNEHRLNQLLSIAEECETLDRLKQLVDSGRIFTAYNGFEPSGRIHIAQALITVMNTNKIIECGGQMIIYIAD
WFAKMNLKMNGDINKIRELGRYFIEVFKACGINLDGTRFIWASEFIASNPSYIERMLDIAEFSTISRVKRCCQIMGRNE
SDCLKASQIFYPCMQAADVFELVPEGIDICQLGIDQRKVNMLAIEYANDRGLKIPISLSHHMLMSLSGPKKKMSKSD
PQGAIFMDDTEQEVSEKISRAYCTDEIFDNPIFEYIKYLLLRWFGTLNLCGKIYTDIESIQEDFSSMNKRELKTDVANYI
NTIIDLVREHFKKPELSELLSNVKSYQQPSK

Mimivirus sp. SH

MNQFKELLTNVKNISLIYNDNDHHVMFTMKSHLSYFLTRFKNSDVVKIIEKDNLEYFIFDEITSDNYLDIYDKFIKQY
QIYDLDYDNFIDQRVQNLLSISEECDTIDRLKELVESFKNFTAYNGFEPSGRIHIAQALITVMNTNTIIKNGGSMIIYIA
DWFAQMNHKMGGDLEKIRDVGKYFIEVFKACGIDMSGTKFIWASEFFNDNSTKYFERMLHIANQTSLARAKRCCQ
IMGRREGDELSSSQIIYPCMQVADIFELNPNGIDICQLGVDQRKVNMLAIEYAKRNNLKTPIILSHHMLMGLKGSKN
KMSKSDPENAIFMEDSREDIFKKIYKAYCTDAITDNPIYEYIKYILLRWFDTVTLCGQKYNNIDDINKDFATMNKKDL
KNNVADYIDNIIEPVRRHFQDPQMAELYSRVRSYTN

Moumouvirus australiensis

MEQQTLSVEEKFTRLISISEECETPDRLVELINTGKKFTAYNGFEPSGRIHIAQALITVMNTNEIISCGGTMIIYIADLFA
KMNKKMDSDLDKIKDVGLYFIEVFKACGINMSETKFIWSSDLFNKNPEKYFTRLLDISEQTSLSRSKRCCQIMGRKEG
DELSVSQIIYPCMQVADIFELEPEGIDICQLGVDQRKVNMLALEYAKKNGLKVPIILSHHMLMGLGGPKNKMSKSDP
KSAIFMEDSYEEIKEKILRAFCTDDTKDNPIFEYIKYILFRWYGKLELCGKNYNNMDEIEKDFHDMNKRELKNNVAE
YINKIIEPVRIHFQQPHLKDLLEKVSSYRVTK

Niemeyer virus-1

MENTDHTNNEHRLTQLLSIAEECETLDRLKQLVDSGRIFTAYNGFEPSGRIHIAQALITVMNTNNIIECGGQMIIYIAD
WFAKMNLKMNGDINKIRELGRYFIEVFKACGINLDGTRFIWASEFIASNPSYIERMLDIAEFSTISRVKRCCQIMGRNE
SDCLKASQIFYPCMQAADVFELVPEGIDICQLGIDQRKVNMLAIEYANDRGLKIPISLSHHMLMSLSGPKKKMSKSD
PQGAIFMDDTEQEVSEKISRAYCTDETFDNPIFEYIKYLLLRWFGTLNLCGKIYTDIESIQEDFSSMNKRELKTDVANYI
NTIIDLVREHFKKPELSELLSNVKSYQQPSK

Niemeyer virus-2

MENTDYSNNEHRLNQLLLIAEECETLDRLKQLVDSGKIFTAYNGFEPSGRIHIAQALITVMNTNKIIECGGQMIIYIA
DWFAKMNLKMNGDIEKIRELGRYFIEVFKACGINLGGTKFIWASEFIASNPSYIERMLDIAEFSTLSRVKRCCQIMGR
NESDCLKASQIFYPCMQAADVFELVPGGIDICQLGIDQRKVNMLAIEYANDRGLKIPISLSHHMLMSLNGPKKKMS
KSDPQSAIFMDDTEQEINEKISRAYCTDEIFDNPIFEYIKYLLLRWFGTLNLCGKVYTDIKSIEQDFSSMNKRELKTDVA

NYINIIIDLVREHFKKPELSDLLSNIKSYQQSSK

Orpheovirus IHUMI-LCC2

MNTYNLITRNLQEVVGNDELLKKIDNGEDIKVYWGTAITGNPHLGYFVPILKIADLLNAGCKVTILLADLHGYLDN
LKTDWDLLSLRIEWYELIIKEMLKRVGVSLENLKFVTGSTFQLSKEYTLDMYKLSALVTTTAMQHASAEVVKQSKNP
LLSGLLYPILQALDEKYLGVNVQLGGIDQRKIFMFAREILSEVDYPKSIHLMNPLVPGLGKPVKGNKGKNEGNDIEG
KMSSSDKNSKIDFSDSDEVIKDKMKKAYSVDGQVEGNCLMSMCKLILFPFLELEGRKFVVEREEKYGGDVHFDNYN
SIEETFGKKELASMDLKVAIAKEIIKLVNPIRKIVNDNVELMKSAYPTEKN

Pandoravirus dulcis

MISDDGDDNSGGKEAAHLPVGNIDPDQGDVEQRYALVVRDLDEVIGDPAEIKAIMAERPLIIYWGTAPTGNPHLGY
FVPIFKLADFLQAGCRVKILFADVHAHLDNGKTPWDLVEHRCRWYEFAIKAMLQHIGVPLGGLEFVRGTEYQCGG
AYALDLYRLAAHVRTGTAQKAAAQVVKMDRNPLLSNVLYPLMQALDEQHLDVDAQFGGRDQRKIFAFARDHLP
VLGYRKRFHLLNPLVPGLTESGKMSASEAASKIGLDDPDEVIRAKIRRAYSVDGCAEGNGLLAILRFVLWRWLEPAG
LPFVVTRPSEYGGPQTFATYAEVEAAFVRAGVPLVEGGGERLFSVDLKPAVADLLCEFLAPLRGVLAARADLFAAAY
PV

Pandoravirus japonicus

MIDDDDDGGKETAHLPTDDGALDQDDVEQRYALVVRDLDEVIGEPAEIKAIMAERPLAVYWGTAPTGNPHLGYFV
PIFKLADFLQAGCRVKILFADVHAHLDNAKTPWDLVEHRCRWYEFAIKAMLQHIGVPLDRLEFVRGSDYQHGTAY
TLDLLRLTARVTVATAQKAAAQVVKMERNPLLSNVIYPLMQALDEEHLDVDAQFGGRDQRKIFAFARDHLPALGY
RKRVHLLNPLVPGLTKSGKMSASEAASKIGLDDPDEVIRAKIRQAYSVDGCAEGNGLLAILRFVLWRWLEPAGLPFV
VTRPPEYGGPQTFATYAEVEAAFVRAGVPPAEGDGERLFSVDLKPAVADLLCEFLAPLRGVLAARADLLHAAYPPPS

Pandoravirus macleodensis

MDDARDQILQDDKNRAFGASSHTSSSTSTSIQQPAGIIGLPRSRDNIADDDVEYRYGLMTRDLEEVIGDPAEIKAVM
AERPLVIYWGTAPTGKPHLGYFVPIFKLADFLQAGCRVKILFANVHAHLDNGKTPWGLVEHRCQWYEFAIKAMLQ
HIGVPLDRLEFVRGTEYQCSSAYTLDLYRLTAQVSTGAAQKAAAQVVKMDRNPLLSNVLYPLMQALDEHHLDVDA
QFGGRDQRKIFAFARDHLPALGYRKRFHLLNPLVPGLTKDGKMSASEAASKIDLDDAEEAIRAKIRRAYSVDGRAEG
NGLLAILRFVLWRWLEPAGLPFVVTRPAQYGGPLTFATYADVEAAFVRSHTPSPSTDVDGNNNADGGERLCSADLK
PAIADLLCEFLAPLRGVLGARADLLRAAYPPMPH

Pandoravirus salinus

MMDDTDHQHREKTEKSVHSQGGNGSPTPIGVDSTARQQTDGDDVEHRYGLVVRDLDEVIGDPADIKAIMAERPLC
IYWGTAPTGKPHLGYFVPIFKLADFLQAGCRVRILFADVHAHLDNGKTPWDLVEHRCRWYEFAIKAMLQHIGVPL
DRLEFVRGSDYQHGAAYTLDLLRLTARVTVGTAQKAAAQVVKMDRNPPLSNLVYPLMQALDEQHLDVDAQFGG
RDQRKIFAFARDHLPALGYRKRIHLLNPLVPGLTKDGKMSASEAASKIDLDDCDEVIRAKIRRAYSVDGRAEGNGLL
AILRFILWRWLEPAGLPFVVTRPREYGGPLTFATYADVEAAFVRAGVPLSETGGDGDRLFSVDLKPAVADLLCEFLAP
LRGVLAARADLLATAYPSAP

Samba virus

MENTDHTNNEHRLTQLLSIAEECETLDRLKQLVDSGRIFTAYNGFEPSGRIHIAQALITVMNTNNIIECGGQMIIYIAD
WFAKMNLKMNGDINKIRELGRYFIEVFKACGINLDGTRFIWASEFIASNPSYIERMLDIAEFSTISRVKRCCQIMGRNE
SDCLKASQIFYPCMQAADVFELVPEGIDICQLGIDQRKVNMLAIEYANDRGLKIPISLSHHMLMSLSGPKKKMSKSD
PQGAIFMDDTEQEVSEKISRAYCTDETFDNPIFEYIKYLLLRWFGTLNLCGKIYTDIESIQEDFSSMNKRELKTDVANYI
NTIIDLVREHFKKPELSELLSNVKSYQQPSK

Saudi moumouvirus

MEQQILSTEEKITRLISICEECETPDRLEELIKSGKKFTAYNGFEPSGRIHIAQALITVMNTNEIISCGGRMIIYIADLFAK
MNKKMDSDLDKIKDVGLYFIEVFKACGINMSETKFIWSSDLFNKNPEKYFTRLLDISEETSLSRSKRCCQIMGRKDGD

ELSVSQIIYPCMQVADIFELEPEGIDICQLGVDQRKVNMLALEYAKKNGLKVPVILSHHMLMGLGGPKNKMSKSDP
KSAIFMEDSYEEIKEKILRAFCTDDINENPIFEYIKHILFRWYGKLELCGKNYDNMSDIEKDFCDMNKRELKNNVAEY
INKIIEPVRIHFQQPHLKNLLDKVSSYRVTK

Terrestrivirus sp.

MNPQEQYDLISHRISPDDIVGKSYLMEKLQAGKQLKGYVGFAPTGRCHIGYASLLLKVAQCLDAGCEIIILLADVHA
FLDARKSGLEDEAKTEYYKQMITELLRLLNADLSKVKFVKGSEYQYSKPYMQDLLRLLNTISISKAKHAGADVVKQ
AEDPLLSSLVYPLMQSLDETHLDPDLDFELSGTDQRHIFMFSVDYVNKSKNKKMTYIMNQLVPGLSKESLRNPETKV
VTSQKMSSSDSVGKLDLLDTPKEILNKLKKAYCLEGDAEDNTVLVMCKHIIFPVLERLNKQFVIARDEKYGGNLVIN
SHDELYALFADKSIHPSDLKKSVANIITDLFEPIRQHFSTNDMVKLLEQAYGK

Tupanvirus deep ocean

MEIPDDKLSLITSRLQEIIGLDELQKIIQSRPLKIYFGTAPTGRIHIGYLVPLLKIADFLKAGCEVKILFADLHAVLDNLKS
TMQQVEFRTSYYEKMIKSILKNLNVPLDKLTFVRGSSFQLSQAYTMDMYKISTLATLHDAKKAGAEVVKQTDNPKM
SGLLYPILQALDEQYLDVDAQFGGVDQRKLFTFAADVLPLIGYKKRIHLMNPMLTAINAMPKDSNENTNISEIKMSS
SDLNSKIDMLDSKNEIKKKINRAYCLEGDLSFNPLMELMKHVTFPLLNNCGSSIFKINRKEIHGGPLSFSNYADLEKD
FVEKKLHPQDLKTGIIDCLNLFMEPIRAEFNDKESQSLIKQAYN

Tupanvirus soda lake

MEVVQDKISLITSRLQEVIGIDELQNIIKSRPLKIYWGTAPTGKVHIGYLVPLLKIADFLKSGCEVKILFADLHAVLDNL
KSTSQQVEYRTIYYEKMIKAILKNLNVSLEKLTFVKGSSFQLTQSYTMDMYKISTLATLHDAKKAGAEVVKQTDNPK
MSGLLYPILQALDEQYLDVDAQFGGIDQRKLFTFAADVLPLIGYNKRIHLMNPMLTAINAMPKNINEDTNLSEVKM
SSSDLNSKIDMMDSKNEVKKKINRAYCLEGDLSFNPLMELMKLVIFPLLDNCNINIFTINRHEKYGGPLNYSNFEELE
KDFVQKTLHPQDLKMGIIDCLNNFMDPIRQEFSDKETQQLIKKAYN

Supplementary Sequence 2 The plasmid sequences used in this work

pEvole-MjTyrRS(wt)

*TCCTGAAAATCTCGATAACTCAAAAAATACGCCCGGTAGTGATCTTATTTCATTATGGTGAAAGTTGGAACCTCTTAC
GTGCCGATCAACGTCTCATTTTCGCCAAAAGTTGGCCCAGGGCTTCCCGGTATCAACAGGGACACCAGGATTTATTT
ATTCTGCGAAGTGATCTTCCGTCACAGGTATTTATTCGGCGCAAAGTGCGTCGGGTGATGCTGCCAACTTACTGATT
TAGTGTATGATGGTGTTTTTGAGGTGCTCCAGTGGCTTCTGTTTCTATCAGCTGTCCCTCCTGTTCAGCTACTGACGG
GGTGGTGCGTAACGGCAAAAGCACCGCCGGACATCAGCGCTAGCGGAGTGTATACTGGCTTACTATGTTGGCACTG
ATGAGGGTGTCAGTGAAGTGCTTCATGTGGCAGGAGAAAAAAGGCTGCACCGGTGCGTCAGCAGAATATGTGATA
CAGGATATATTCCGCTTCCTCGCTCACTGACTCGCTACGCTCGGTCGTTCGACTGCGGCGAGCGGAAATGGCTTACG
AACGGGGCGGAGATTTCCTGGAAGATGCCAGGAAGATACTTAACAGGGAAGTGAGAGGGCCGCGGCAAAGCCGTT
TTTCCATAGGCTCCGCCCCCCTGACAAGCATCACGAAATCTGACGCTCAAATCAGTGGTGGCGAAACCCGACAGGA
CTATAAAGATACCAGGCGTTTCCCCCTGGCGGCTCCCTCGTGCGCTCTCCTGTTCCTGCCTTTCGGTTTACCGGTGTC
ATTCCGCTGTTATGGCCGCGTTTGTCTCATTCCACGCCTGACACTCAGTTCCGGGTAGGCAGTTCGCTCCAAGCTGG
ACTGTATGCACGAACCCCCCGTTCAGTCCGACCGCTGCGCCTTATCCGGTAACTATCGTCTTGAGTCCAACCCGGAA
AGACATGCAAAAGCACCACTGGCAGCAGCCACTGGTAATTGATTTAGAGGAGTTAGTCTTGAAGTCATGCGCCGGT
TAAGGCTAAACTGAAAGGACAAGTTTTGGTGACTGCGCTCCTCCAAGCCAGTTACCTCGGTTCAAAGAGTTGGTAG
CTCAGAGAACCTTCGAAAAACCGCCCTGCAAGGCGGTTTTTTCGTTTTCAGAGCAAGAGATTACGCGCAGACCAAA
ACGATCTCAAGAAGATCATCTTATTAATCAGATAAAATATTTCTAGATTTCAGTGCAATTTATCTCTTCAAATGTAGC
ACCTGAAGTCAGCCCCATACGATATAAGTTGTAATTCTCATGTTTGACAGCTTATCATCGATAAGCTTGGTACCCAAT
TATGACAACTTGACGGCTACATCATTCACTTTTTCTTCACAACCGGCACGGAACTCGCTCGGGCTGGCCCCGGTGCA
TTTTTTAAATACCCGCGAGAAATAGAGTTGATCGTCAAAACCAACATTGCGACCGACGGTGGCGATAGGCATCCGG*

GTGGTGCTCAAAAGCAGCTTCGCCTGGCTGATACGTTGGTCCTCGCGCCAGCTTAAGACGCTAATCCCTAACTGCTG
GCGGAAAAGATGTGACAGACGCGACGGCGACAAGCAAACATGCTGTGCGACGCTGGCGATATCAAAATTGCTGTC
TGCCAGGTGATCGCTGATGTACTGACAAGCCTCGCGTACCCGATTATCCATCGGTGGATGGAGCGACTCGTTAATCG
CTTCCATGCGCCGCAGTAACAATTGCTCAAGCAGATTTATCGCCAGCAGCTCCGAATAGCGCCCTTCCCCTTGCCCG
GCGTTAATGATTTGCCCAAACAGGTCGCTGAAATGCGGCTGGTGCGCTTCATCCGGGCGAAAGAACCCCGTATTGG
CAAATATTGACGGCCAGTTAAGCCATTCATGCCAGTAGGCGCGCGGACGAAAGTAAACCCACTGGTGATACCATTC
GCGAGCCTCCGGATGACGACCGTAGTGATGAATCTCTCCTGGCGGGAACAGCAAAATATCACTCGGTCGGCAAACA
AATTCTCGTCCCTGATTTTTCACCACCCCCTGACCGCGAATGGTGAGATTGAGAATATAACCTTTCATTCCCAGCGG
TCGGTCGATAAAAAAATCGAGATAACCGTTGGCCTCAATCGGCGTTAAACCCGCCACCAGATGGGCATTAAACGAG
TATCCCGGCAGCAGGGGATCATTTTGCGCTTCAGCCATACTTTTCATACTCCCGCCATTCAGAGAAGAAACCAATTG
TCCATATTGCATCAGACATTGCCGTCACTGCGTCTTTTACTGGCTCTTCTCGCTAACCAAACCGGTAACCCCGCTTAT
TAAAAGCATTCTGTAACAAAGCGGGACCAAAGCCATGACAAAAACGCGTAACAAAAGTGTCTATAATCACGGCAG
AAAAGTCCACATTGATTATTTGCACGGCGTCACACTTTGCTATGCCATAGCATTTTTATCCATAAGATTAGCGGATCC
TACCTGACGCTTTTTATCGCAACTCTCTACTGTTTCTCCATACCCGTTTTTTTGGGCTAACAGGAGGAATTAGATCTAT
GGACGAATTTGAAATGATAAAGAGAAACACATCTGAAATTATCAGCGAGGAAGAGTTAAGAGAGGTTTTAAAAAA
AGATGAAAAATCTGCTTACATAGGTTTTGAACCAAGTGGTAAAATACATTTAGGGCATTATCTCCAAATAAAAAAGA
TGATTGATTTACAAAATGCTGGATTTGATATAATTATATTGTTGGCTGATTTACACGCCTATTTAAACCAGAAAGGAG
AGTTGGATGAGATTAGAAAAATAGGAGATTATAACAAAAAAGTTTTTGAAGCAATGGGGTTAAAGGCAAAATATGT
TTATGGAAGTGAATTCCAGCTTGATAAGGATTATACACTGAATGTCTATAGATTGGCTTTAAAAACTACCTTAAAAAG
AGCAAGAAGGAGTATGGAACTTATAGCAAGAGAGGATGAAAATCCAAAGGTTGCTGAAGTTATCTATCCAATAATG
CAGGTTAATGATATTCATTATTTAGGCGTTGATGTTGCAGTTGGAGGGATGGAGCAGAGAAAAATACACATGTTAGC
AAGGGAGCTTTTACCAAAAAAGGTTGTTTGTATTCACAACCCTGTCTTAACGGGTTTGGATGGAGAAGGAAAGATG
AGTTCTTCAAAAGGGAATTTTATAGCTGTTGATGACTCTCCAGAAGAGATTAGGGCTAAGATAAAGAAAGCATACTG
CCCAGCTGGAGTTGTTGAAGGAAATCCAATAATGGAGATAGCTAAATACTTCCTTGAATATCCTTTAACCATAAAAA
GGCCAGAAAAATTTGGTGGAGATTTGACAGTTAATAGCTATGAGGAGTTAGAGAGTTTATTTAAAAATAAGGAATTG
CATCCAATGGATTTAAAAAATGCTGTAGCTGAAGAACTTATAAAGATTTTAGAGCCAATTAGAAAGAGATTATAAGT
CGACCATCATCATCATCATCATTGAGTTTAAACGGTCTCCAGCTTGGCTGTTTTGGCGGATGAGAGAAGATTTTCAG
CCTGATACAGATTAAATCAGAACGCAGAAGCGGTCTGATAAAACAGAATTTGCCTGGCGGCAGTAGCGCGGTGGTC
CCACCTGACCCCATGCCGAACTCAGAAGTGAAACGCCGTAGCGCCGATGGTAGTGTGGGGTCTCCCCATGCGAGAG
TAGGGAACTGCCAGGCATCAAATAAAACGAAAGGCTCAGTCGAAAGACTGGGCCTTGTTTGTGAGCTCCCGGTCAT
CACTGCAGTTTCAAACGCTAAATTGCCTGATGCGCTACGCTTATCAGGCCTACATGATCTCTGCAATATATTGAGTTT
GCGTGCTTTTGTAGGCCGGATAAGGCGTTCACGCCGCATCCGGCAAGAAACAGCAAACAATCCAAAACGCCGCGTT
CAGCGGCGTTTTTTCTGCTTTTCTTCGCGAATTAATTCCGCTTCGCAACATGTGAGCACCGGTTTATTGACTACCGGA
AGCAGTGTGACCGCAGCTCAGGGTCGAATTTGCTTTCGAATTTCTGCCATTCATCCGCTTATTATCACTTATTCAGGC
GTAGCACCAGGCGTTTAAGGGCACCAATAACTGCCTTAAAAAAATTACGCCCCGCCCTGCCACTCATCGCAGTACTG
TTGTAATTCATTAAGCATTCTGCCGACATGGAAGCCATCACAGACGGCATGATGAACCTGAATCGCCAGCGGCATC
AGCACCTTGTCGCCTTGCGTATAATATTTGCCCATGGTGAAAACGGGGGCGAAGAAGTTGTCCATATTGGCCACGTT
TAAATCAAAACTGGTGAAACTCACCCAGGGATTGGCTGAGACGAAAAACATATTCTCAATAAACCCTTTAGGGAAA
TAGGCCAGGTTTTCACCGTAACACGCCACATCTTGCGAATATATGTGTAGAAACTGCCGGAAATCGTCGTGGTATTC
ACTCCAGAGCGATGAAAACGTTTCAGTTTGCTCATGGAAAACGGTGTAACAAGGGTGAACACTATCCCATATCACC
AGCTCACCGTCTTTCATTGCCATACGGAATTCCGGATGAGCATTCATCAGGCGGGCAAGAATGTGAATAAAGGCCG
GATAAAACTTGTGCTTATTTTTCTTTACGGTCTTTAAAAAGGCCGTAATATCCAGCTGAACGGTCTGGTTATAGGTAC
ATTGAGCAACTGACTGAAATGCCTCAAAATGTTCTTTACGATGCCATTGGGATATATCAACGGTGGTATATCCAGTG
ATTTTTTTCTCCATTTTAGCTTCCTTAGC

pEvole-MjTyrRS(wt)-Loop1

*TCCTGAAAATCTCGATAACTCAAAAAATACGCCCGGTAGTGATCTTATTTCATTATGGTGAAAGTTGGAACCTCTTAC
GTGCCGATCAACGTCTCATTTTCGCCAAAAGTTGGCCCAGGGCTTCCCGGTATCAACAGGGACACCAGGATTTATTT
ATTCTGCGAAGTGATCTTCCGTCACAGGTATTTATTCGGCGCAAAGTGCGTCGGGTGATGCTGCCAACTTACTGATT
TAGTGTATGATGGTGTTTTTGAGGTGCTCCAGTGGCTTCTGTTTCTATCAGCTGTCCCTCCTGTTCAGCTACTGACGG
GGTGGTGCGTAACGGCAAAAGCACCGCCGGACATCAGCGCTAGCGGAGTGTATACTGGCTTACTATGTTGGCACTG
ATGAGGGTGTCAGTGAAGTGCTTCATGTGGCAGGAGAAAAAAGGCTGCACCGGTGCGTCAGCAGAATATGTGATA
CAGGATATATTCCGCTTCCTCGCTCACTGACTCGCTACGCTCGGTCGTTCGACTGCGGCGAGCGGAAATGGCTTACG
AACGGGGCGGAGATTTCCTGGAAGATGCCAGGAAGATACTTAACAGGGAAGTGAGAGGGCCGCGGCAAAGCCGTT
TTTCCATAGGCTCCGCCCCCCTGACAAGCATCACGAAATCTGACGCTCAAATCAGTGGTGGCGAAACCCGACAGGA
CTATAAAGATACCAGGCGTTTCCCCCTGGCGGCTCCCTCGTGCGCTCTCCTGTTCCTGCCTTTCGGTTTACCGGTGTC
ATTCCGCTGTTATGGCCGCGTTTGTCTCATTCCACGCCTGACACTCAGTTCCGGGTAGGCAGTTCGCTCCAAGCTGG
ACTGTATGCACGAACCCCCCGTTCAGTCCGACCGCTGCGCCTTATCCGGTAACTATCGTCTTGAGTCCAACCCGGAA
AGACATGCAAAAGCACCACTGGCAGCAGCCACTGGTAATTGATTTAGAGGAGTTAGTCTTGAAGTCATGCGCCGGT
TAAGGCTAAACTGAAAGGACAAGTTTTGGTGACTGCGCTCCTCCAAGCCAGTTACCTCGGTTCAAAGAGTTGGTAG
CTCAGAGAACCTTCGAAAAACCGCCCTGCAAGGCGGTTTTTTCGTTTTCAGAGCAAGAGATTACGCGCAGACCAAA
ACGATCTCAAGAAGATCATCTTATTAATCAGATAAAATATTTCTAGATTTCAGTGCAATTTATCTCTTCAAATGTAGC
ACCTGAAGTCAGCCCCATACGATATAAGTTGTAATTCTCATGTTTGACAGCTTATCATCGATAAGCTTGGTACCCAAT
TATGACAACTTGACGGCTACATCATTCACTTTTTCTTCACAACCGGCACGGAACTCGCTCGGGCTGGCCCCGGTGCA
TTTTTTAAATACCCGCGAGAAATAGAGTTGATCGTCAAAACCAACATTGCGACCGACGGTGGCGATAGGCATCCGG
GTGGTGCTCAAAAGCAGCTTCGCCTGGCTGATACGTTGGTCCTCGCGCCAGCTTAAGACGCTAATCCCTAACTGCTG
GCGGAAAAGATGTGACAGACGCGACGGCGACAAGCAAACATGCTGTGCGACGCTGGCGATATCAAAATTGCTGTC
TGCCAGGTGATCGCTGATGTACTGACAAGCCTCGCGTACCCGATTATCCATCGGTGGATGGAGCGACTCGTTAATCG
CTTCCATGCGCCGCAGTAACAATTGCTCAAGCAGATTTATCGCCAGCAGCTCCGAATAGCGCCCTTCCCCTTGCCCG
GCGTTAATGATTTGCCCAAACAGGTCGCTGAAATGCGGCTGGTGCGCTTCATCCGGGCGAAAGAACCCCGTATTGG
CAAATATTGACGGCCAGTTAAGCCATTCATGCCAGTAGGCGCGCGGACGAAAGTAAACCCACTGGTGATACCATTC
GCGAGCCTCCGGATGACGACCGTAGTGATGAATCTCTCCTGGCGGGAACAGCAAAATATCACTCGGTCGGCAAACA
AATTCTCGTCCCTGATTTTTCACCACCCCCTGACCGCGAATGGTGAGATTGAGAATATAACCTTTCATTCCCAGCGG
TCGGTCGATAAAAAAATCGAGATAACCGTTGGCCTCAATCGGCGTTAAACCCGCCACCAGATGGGCATTAAACGAG
TATCCCGGCAGCAGGGGATCATTTTGCGCTTCAGCCATACTTTTCATACTCCCGCCATTCAGAGAAGAAACCAATTG
TCCATATTGCATCAGACATTGCCGTCACTGCGTCTTTTACTGGCTCTTCTCGCTAACCAAACCGGTAACCCCGCTTAT
TAAAAGCATTCTGTAACAAAGCGGGACCAAAGCCATGACAAAAACGCGTAACAAAAGTGTCTATAATCACGGCAG
AAAAGTCCACATTGATTATTTGCACGGCGTCACACTTTGCTATGCCATAGCATTTTTATCCATAAGATTAGCGGATCC
TACCTGACGCTTTTTATCGCAACTCTCTACTGTTTCTCCATACCCGTTTTTTTGGGCTAACAGGAGGAATTAGATCTAT
GGACGAATTTGAAATGATAAAGAGAAACACATCTGAAATTATCAGCGAGGAAGAGTTAAGAGAGGTTTTAAAAAA
AGATGAAAAATCTGCTTACATAGGTTTTGAACCAAGTGGTAAAATACATTTAGGGCATTATCTCCAAATAAAAAAGA
TGATTGATTTACAAAATGCTGGATTTGATATAATTATATTGTTGGCTGATTTACACGCCTATTTAAACCAGAAAGGAG
AGTTGGATGAGATTAGAAAAATAGGAGATTATAACAAAAAAGTTTTTGAAGCAATGGGGTTAAAGGCAAAATATGT
TTATGGAAGTGAATTCCAGCTTGATAAGGATTATACACTGAATGTCTATAGATTGGCTTTAAAAACTACCTTAAAAAG
AGCAAGAAGGAGTATGGAACTTATAGCAAGAGAGGATGAAAATCCAAAGGTTGCTGAAGTTATCTATCCAATAATG
CAGGTTAATGATATTCATTATTTAGGCGTTGATGTTGCAGTTGGAGGGATGGAGCAGAGAAAAATACACATGTTAGC
AAGGGAGCTTTTACCAAAAAAGGTTGTTTGTATTCACAACCCTGTCTTAACGGGTTTGGATGGAGAAGGAAAGATG
AGTTCTTCAAAAGGGAATTTTATAGCTGTTGATGACTCTCCAGAAGAGATTAGGGCTAAGATAAAGAAAGCATACTG
CCCAGCTGGAGTTGTTGAAGGAAATCCAATAATGGAGATAGCTAAATACTTCCTTGAATATCCTTTAACCCTGTGCG*

GCTTGACAGTTAATAGCTATGAGGAGTTAGAGAGTTTATTTAAAAATAAGGAATTGCATCCAATGGATTTAAAAAT
GCTGTAGCTGAAGAACTTATAAAGATTTTAGAGCCAATTAGAAAGAGATTATAAGTCGACCATCATCATCATCATCA
TTGAGTTTAAACGGTCTCCAGCTTGGCTGTTTTGGCGGATGAGAGAAGATTTTCAGCCTGATACAGATTAAATCAGA
ACGCAGAAGCGGTCTGATAAAACAGAATTTGCCTGGCGGCAGTAGCGCGGTGGTCCCACCTGACCCCATGCCGAA
CTCAGAAGTGAAACGCCGTAGCGCCGATGGTAGTGTGGGGTCTCCCCATGCGAGAGTAGGGAACTGCCAGGCATC
AAATAAAACGAAAGGCTCAGTCGAAAGACTGGGCCTTGTTTGTGAGCTCCCGGTCATCACTGCAGTTTCAAACGCT
AAATTGCCTGATGCGCTACGCTTATCAGGCCTACATGATCTCTGCAATATATTGAGTTTGCGTGCTTTTGTAGGCCGG
ATAAGGCGTTCACGCCGCATCCGGCAAGAAACAGCAAACAATCCAAAACGCCGCGTTCAGCGGCGTTTTTTCTGCT
TTTCTTCGCGAATTAATTCCGCTTCGCAACATGTGAGCACCGGTTTATTGACTACCGGAAGCAGTGTGACCGCAGCT
CAGGGTCGAATTTGCTTTCGAATTTCTGCCATTCATCCGCTTATTATCACTTATTCAGGCGTAGCACCAGGCGTTTAA
GGGCACCAATAACTGCCTTAAAAAAATTACGCCCCGCCCTGCCACTCATCGCAGTACTGTTGTAATTCATTAAGCAT
TCTGCCGACATGGAAGCCATCACAGACGGCATGATGAACCTGAATCGCCAGCGGCATCAGCACCTTGTCGCCTTGC
GTATAATATTTGCCCATGGTGAAAACGGGGGCGAAGAAGTTGTCCATATTGGCCACGTTTAAATCAAAACTGGTGAA
ACTCACCCAGGGATTGGCTGAGACGAAAAACATATTCTCAATAAACCCTTTAGGGAAATAGGCCAGGTTTTCACCGT
AACACGCCACATCTTGCGAATATATGTGTAGAAACTGCCGGAAATCGTCGTGGTATTCACTCCAGAGCGATGAAAA
CGTTTCAGTTTGCTCATGGAAAACGGTGTAACAAGGGTGAACACTATCCCATATCACCAGCTCACCGTCTTTCATTG
CCATACGGAATTCCGGATGAGCATTCATCAGGCGGGCAAGAATGTGAATAAAGGCCGGATAAAACTTGTGCTTATTT
TTCTTTACGGTCTTTAAAAAGGCCGTAATATCCAGCTGAACGGTCTGGTTATAGGTACATTGAGCAACTGACTGAAA
TGCCTCAAAATGTTCTTTACGATGCCATTGGGATATATCAACGGTGGTATATCCAGTGATTTTTTTCTCCATTTTAGCT
TCCTTAGC

pEvole-MjTyrRS(wt)-Loop2

TCCTGAAAATCTCGATAACTCAAAAAATACGCCCGGTAGTGATCTTATTTCATTATGGTGAAAGTTGGAACCTCTTAC
GTGCCGATCAACGTCTCATTTTCGCCAAAAGTTGGCCCAGGGCTTCCCGGTATCAACAGGGACACCAGGATTTATTT
ATTCTGCGAAGTGATCTTCCGTCACAGGTATTTATTCGGCGCAAAGTGCGTCGGGTGATGCTGCCAACTTACTGATT
TAGTGTATGATGGTGTTTTTGAGGTGCTCCAGTGGCTTCTGTTTCTATCAGCTGTCCCTCCTGTTCAGCTACTGACGG
GGTGGTGCGTAACGGCAAAAGCACCGCCGGACATCAGCGCTAGCGGAGTGTATACTGGCTTACTATGTTGGCACTG
ATGAGGGTGTCAGTGAAGTGCTTCATGTGGCAGGAGAAAAAAGGCTGCACCGGTGCGTCAGCAGAATATGTGATA
CAGGATATATTCCGCTTCCTCGCTCACTGACTCGCTACGCTCGGTCGTTCGACTGCGGCGAGCGGAAATGGCTTACG
AACGGGGCGGAGATTTCCTGGAAGATGCCAGGAAGATACTTAACAGGGAAGTGAGAGGGCCGCGGCAAAGCCGTT
TTTCCATAGGCTCCGCCCCCCTGACAAGCATCACGAAATCTGACGCTCAAATCAGTGGTGGCGAAACCCGACAGGA
CTATAAAGATACCAGGCGTTTCCCCCTGGCGGCTCCCTCGTGCGCTCTCCTGTTCCTGCCTTTCGGTTTACCGGTGTC
ATTCCGCTGTTATGGCCGCGTTTGTCTCATTCCACGCCTGACACTCAGTTCCGGGTAGGCAGTTCGCTCCAAGCTGG
ACTGTATGCACGAACCCCCCGTTCAGTCCGACCGCTGCGCCTTATCCGGTAACTATCGTCTTGAGTCCAACCCGGAA
AGACATGCAAAAGCACCACTGGCAGCAGCCACTGGTAATTGATTTAGAGGAGTTAGTCTTGAAGTCATGCGCCGGT
TAAGGCTAAACTGAAAGGACAAGTTTTGGTGACTGCGCTCCTCCAAGCCAGTTACCTCGGTTCAAAGAGTTGGTAG
CTCAGAGAACCTTCGAAAAACCGCCCTGCAAGGCGGTTTTTTCGTTTTCAGAGCAAGAGATTACGCGCAGACCAAA
ACGATCTCAAGAAGATCATCTTATTAATCAGATAAAATATTTCTAGATTTCAGTGCAATTTATCTCTTCAAATGTAGC
ACCTGAAGTCAGCCCCATACGATATAAGTTGTAATTCTCATGTTTGACAGCTTATCATCGATAAGCTTGGTACCCAAT
TATGACAACTTGACGGCTACATCATTCACTTTTTCTTCACAACCGGCACGGAACTCGCTCGGGCTGGCCCCGGTGCA
TTTTTTAAATACCCGCGAGAAATAGAGTTGATCGTCAAAACCAACATTGCGACCGACGGTGGCGATAGGCATCCGG
GTGGTGCTCAAAAGCAGCTTCGCCTGGCTGATACGTTGGTCCTCGCGCCAGCTTAAGACGCTAATCCCTAACTGCTG
GCGGAAAAGATGTGACAGACGCGACGGCGACAAGCAAACATGCTGTGCGACGCTGGCGATATCAAAATTGCTGTC
TGCCAGGTGATCGCTGATGTACTGACAAGCCTCGCGTACCCGATTATCCATCGGTGGATGGAGCGACTCGTTAATCG
CTTCCATGCGCCGCAGTAACAATTGCTCAAGCAGATTTATCGCCAGCAGCTCCGAATAGCGCCCTTCCCCTTGCCCG

GCGTTAATGATTTGCCCAAACAGGTCGCTGAAATGCGGCTGGTGCGCTTCATCCGGGCGAAAGAACCCCGTATTGG
CAAATATTGACGGCCAGTTAAGCCATTCATGCCAGTAGGCGCGCGGACGAAAGTAAACCCACTGGTGATACCATTC
GCGAGCCTCCGGATGACGACCGTAGTGATGAATCTCTCCTGGCGGGAACAGCAAAATATCACTCGGTCGGCAAACA
AATTCTCGTCCCTGATTTTTCACCACCCCCTGACCGCGAATGGTGAGATTGAGAATATAACCTTTCATTCCCAGCGG
TCGGTCGATAAAAAAATCGAGATAACCGTTGGCCTCAATCGGCGTTAAACCCGCCACCAGATGGGCATTAAACGAG
TATCCCGGCAGCAGGGGATCATTTTGCGCTTCAGCCATACTTTTCATACTCCCGCCATTCAGAGAAGAAACCAATTG
TCCATATTGCATCAGACATTGCCGTCACTGCGTCTTTTACTGGCTCTTCTCGCTAACCAAACCGGTAACCCCGCTTAT
TAAAAGCATTCTGTAACAAAGCGGGACCAAAGCCATGACAAAAACGCGTAACAAAAGTGTCTATAATCACGGCAG
AAAAGTCCACATTGATTATTTGCACGGCGTCACACTTTGCTATGCCATAGCATTTTTATCCATAAGATTAGCGGATCC
TACCTGACGCTTTTTATCGCAACTCTCTACTGTTTCTCCATACCCGTTTTTTTGGGCTAACAGGAGGAATTAGATCTAT
GGACGAATTTGAAATGATAAAGAGAAACACATCTGAAATTATCAGCGAGGAAGAGTTAAGAGAGGTTTTAAAAAA
AGATGAAAAATCTGCTTACATAGGTTTTGAACCAAGTGGTAAAATACATTTAGGGCATTATCTCCAAATAAAAAAGA
TGATTGATTTACAAAATGCTGGATTTGATATAATTATATTGTTGGCTGATTTACACGCCTATTTAAACCAGAAAGGAG
AGTTGGATGAGATTAGAAAAATAGGAGATTATAACAAAAAAGTTTTTGAAGCAATGGGGTTAAAGGCAAAATATGT
TTATGGAAGTGAATTCCAGCTTGATAAGGATTATACACTGAATGTCTATAGATTGGCTTTAAAAACTACCTTAAAAAG
AGCAAGAAGGAGTATGGAACTTATAGCAAGAGAGGATGAAAATCCAAAGGTTGCTGAAGTTATCTATCCAATAATG
CAGGTTAATGATATTCATTATTTAGGCGTTGATGTTGCAGTTGGAGGGATGGAGCAGAGAAAAATACACATGTTAGC
AAGGGAGCTTTTACCAAAAAAGGTTGTTTGTATTCACAACCCTGTCTTAACGGGTTTGGATGGAGAAGGAAAGATG
AGTTCTTCAAAAGGGAATTTTATAGCTGTTGATGACTCTCCAGAAGAGATTAGGGCTAAGATAAAGAAAGCATACTG
CCCAGCTGGAGTTGTTGAAGGAAATCCAATAATGGAGATAGCTAAATACTTCCTTGAATATCCTTTAACCGGAAGTG
GTTTGACAGTTAATAGCTATGAGGAGTTAGAGAGTTTATTTAAAAATAAGGAATTGCATCCAATGGATTTAAAAAAT
GCTGTAGCTGAAGAACTTATAAAGATTTTAGAGCCAATTAGAAAGAGATTATAAGTCGACCATCATCATCATCATCA
TTGAGTTTAAACGGTCTCCAGCTTGGCTGTTTTGGCGGATGAGAGAAGATTTTCAGCCTGATACAGATTAAATCAGA
ACGCAGAAGCGGTCTGATAAAACAGAATTTGCCTGGCGGCAGTAGCGCGGTGGTCCCACCTGACCCCATGCCGAA
CTCAGAAGTGAAACGCCGTAGCGCCGATGGTAGTGTGGGGTCTCCCCATGCGAGAGTAGGGAACTGCCAGGCATC
AAATAAAACGAAAGGCTCAGTCGAAAGACTGGGCCTTGTTTGTGAGCTCCCGGTCATCACTGCAGTTTCAAACGCT
AAATTGCCTGATGCGCTACGCTTATCAGGCCTACATGATCTCTGCAATATATTGAGTTTGCGTGCTTTTGTAGGCCGG
ATAAGGCGTTCACGCCGCATCCGGCAAGAAACAGCAAACAATCCAAAACGCCGCGTTCAGCGGCGTTTTTTCTGCT
TTTCTTCGCGAATTAATTCCGCTTCGCAACATGTGAGCACCGGTTTATTGACTACCGGAAGCAGTGTGACCGCAGCT
CAGGGTCGAATTTGCTTTCGAATTTCTGCCATTCATCCGCTTATTATCACTTATTCAGGCGTAGCACCAGGCGTTTAA
GGGCACCAATAACTGCCTTAAAAAAATTACGCCCCGCCCTGCCACTCATCGCAGTACTGTTGTAATTCATTAAGCAT
TCTGCCGACATGGAAGCCATCACAGACGGCATGATGAACCTGAATCGCCAGCGGCATCAGCACCTTGTCGCCTTGC
GTATAATATTTGCCCATGGTGAAAACGGGGGCGAAGAAGTTGTCCATATTGGCCACGTTTAAATCAAAACTGGTGAA
ACTCACCCAGGGATTGGCTGAGACGAAAAACATATTCTCAATAAACCCTTTAGGGAAATAGGCCAGGTTTTCACCGT
AACACGCCACATCTTGCGAATATATGTGTAGAAACTGCCGGAAATCGTCGTGGTATTCACTCCAGAGCGATGAAAA
CGTTTCAGTTTGCTCATGGAAAACGGTGTAACAAGGGTGAACACTATCCCATATCACCAGCTCACCGTCTTTCATTG
CCATACGGAATTCCGGATGAGCATTCATCAGGCGGGCAAGAATGTGAATAAAGGCCGGATAAAACTTGTGCTTATTT
TTCTTTACGGTCTTTAAAAAGGCCGTAATATCCAGCTGAACGGTCTGGTTATAGGTACATTGAGCAACTGACTGAAA
TGCCTCAAAATGTTCTTTACGATGCCATTGGGATATATCAACGGTGGTATATCCAGTGATTTTTTTCTCCATTTTAGCT
TCCTTAGC

pEvole-MjTyrRS(wt)-Loop3

TCCTGAAAATCTCGATAACTCAAAAAATACGCCCGGTAGTGATCTTATTTCATTATGGTGAAAGTTGGAACCTCTTAC
GTGCCGATCAACGTCTCATTTTCGCCAAAAGTTGGCCCAGGGCTTCCCGGTATCAACAGGGACACCAGGATTTATTT
ATTCTGCGAAGTGATCTTCCGTCACAGGTATTTATTCGGCGCAAAGTGCGTCGGGTGATGCTGCCAACTTACTGATT

TAGTGTATGATGGTGTTTTTGAGGTGCTCCAGTGGCTTCTGTTTCTATCAGCTGTCCCTCCTGTTCAGCTACTGACGG
GGTGGTGCGTAACGGCAAAAGCACCGCCGGACATCAGCGCTAGCGGAGTGTATACTGGCTTACTATGTTGGCACTG
ATGAGGGTGTCAGTGAAGTGCTTCATGTGGCAGGAGAAAAAAGGCTGCACCGGTGCGTCAGCAGAATATGTGATA
CAGGATATATTCCGCTTCCTCGCTCACTGACTCGCTACGCTCGGTCGTTCGACTGCGGCGAGCGGAAATGGCTTACG
AACGGGGCGGAGATTTCCTGGAAGATGCCAGGAAGATACTTAACAGGGAAGTGAGAGGGCCGCGGCAAAGCCGTT
TTTCCATAGGCTCCGCCCCCCTGACAAGCATCACGAAATCTGACGCTCAAATCAGTGGTGGCGAAACCCGACAGGA
CTATAAAGATACCAGGCGTTTCCCCCTGGCGGCTCCCTCGTGCGCTCTCCTGTTCCTGCCTTTCGGTTTACCGGTGTC
ATTCCGCTGTTATGGCCGCGTTTGTCTCATTCCACGCCTGACACTCAGTTCCGGGTAGGCAGTTCGCTCCAAGCTGG
ACTGTATGCACGAACCCCCCGTTCAGTCCGACCGCTGCGCCTTATCCGGTAACTATCGTCTTGAGTCCAACCCGGAA
AGACATGCAAAAGCACCACTGGCAGCAGCCACTGGTAATTGATTTAGAGGAGTTAGTCTTGAAGTCATGCGCCGGT
TAAGGCTAAACTGAAAGGACAAGTTTTGGTGACTGCGCTCCTCCAAGCCAGTTACCTCGGTTCAAAGAGTTGGTAG
CTCAGAGAACCTTCGAAAAACCGCCCTGCAAGGCGGTTTTTTCGTTTTCAGAGCAAGAGATTACGCGCAGACCAAA
ACGATCTCAAGAAGATCATCTTATTAATCAGATAAAATATTTCTAGATTTCAGTGCAATTTATCTCTTCAAATGTAGC
ACCTGAAGTCAGCCCCATACGATATAAGTTGTAATTCTCATGTTTGACAGCTTATCATCGATAAGCTTGGTACCCAAT
TATGACAACTTGACGGCTACATCATTCACTTTTTCTTCACAACCGGCACGGAACTCGCTCGGGCTGGCCCCGGTGCA
TTTTTTAAATACCCGCGAGAAATAGAGTTGATCGTCAAAACCAACATTGCGACCGACGGTGGCGATAGGCATCCGG
GTGGTGCTCAAAAGCAGCTTCGCCTGGCTGATACGTTGGTCCTCGCGCCAGCTTAAGACGCTAATCCCTAACTGCTG
GCGGAAAAGATGTGACAGACGCGACGGCGACAAGCAAACATGCTGTGCGACGCTGGCGATATCAAAATTGCTGTC
TGCCAGGTGATCGCTGATGTACTGACAAGCCTCGCGTACCCGATTATCCATCGGTGGATGGAGCGACTCGTTAATCG
CTTCCATGCGCCGCAGTAACAATTGCTCAAGCAGATTTATCGCCAGCAGCTCCGAATAGCGCCCTTCCCCTTGCCCG
GCGTTAATGATTTGCCCAAACAGGTCGCTGAAATGCGGCTGGTGCGCTTCATCCGGGCGAAAGAACCCCGTATTGG
CAAATATTGACGGCCAGTTAAGCCATTCATGCCAGTAGGCGCGCGGACGAAAGTAAACCCACTGGTGATACCATTC
GCGAGCCTCCGGATGACGACCGTAGTGATGAATCTCTCCTGGCGGGAACAGCAAAATATCACTCGGTCGGCAAACA
AATTCTCGTCCCTGATTTTTCACCACCCCCTGACCGCGAATGGTGAGATTGAGAATATAACCTTTCATTCCCAGCGG
TCGGTCGATAAAAAAATCGAGATAACCGTTGGCCTCAATCGGCGTTAAACCCGCCACCAGATGGGCATTAAACGAG
TATCCCGGCAGCAGGGGATCATTTTGCGCTTCAGCCATACTTTTCATACTCCCGCCATTCAGAGAAGAAACCAATTG
TCCATATTGCATCAGACATTGCCGTCACTGCGTCTTTTACTGGCTCTTCTCGCTAACCAAACCGGTAACCCCGCTTAT
TAAAAGCATTCTGTAACAAAGCGGGACCAAAGCCATGACAAAAACGCGTAACAAAAGTGTCTATAATCACGGCAG
AAAAGTCCACATTGATTATTTGCACGGCGTCACACTTTGCTATGCCATAGCATTTTTATCCATAAGATTAGCGGATCC
TACCTGACGCTTTTTATCGCAACTCTCTACTGTTTCTCCATACCCGTTTTTTTGGGCTAACAGGAGGAATTAGATCTAT
GGACGAATTTGAAATGATAAAGAGAAACACATCTGAAATTATCAGCGAGGAAGAGTTAAGAGAGGTTTTAAAAAA
AGATGAAAAATCTGCTTACATAGGTTTTGAACCAAGTGGTAAAATACATTTAGGGCATTATCTCCAAATAAAAAAGA
TGATTGATTTACAAAATGCTGGATTTGATATAATTATATTGTTGGCTGATTTACACGCCTATTTAAACCAGAAAGGAG
AGTTGGATGAGATTAGAAAAATAGGAGATTATAACAAAAAAGTTTTTGAAGCAATGGGGTTAAAGGCAAAATATGT
TTATGGAAGTGAATTCCAGCTTGATAAGGATTATACACTGAATGTCTATAGATTGGCTTTAAAAACTACCTTAAAAAG
AGCAAGAAGGAGTATGGAACTTATAGCAAGAGAGGATGAAAATCCAAAGGTTGCTGAAGTTATCTATCCAATAATG
CAGGTTAATGATATTCATTATTTAGGCGTTGATGTTGCAGTTGGAGGGATGGAGCAGAGAAAAATACACATGTTAGC
AAGGGAGCTTTTACCAAAAAAGGTTGTTTGTATTCACAACCCTGTCTTAACGGGTTTGGATGGAGAAGGAAAGATG
AGTTCTTCAAAAGGGAATTTTATAGCTGTTGATGACTCTCCAGAAGAGATTAGGGCTAAGATAAAGAAAGCATACTG
CCCAGCTGGAGTTGTTGAAGGAAATCCAATAATGGAGATAGCTAAATACTTCCTTGAATATCCTTTAACCGGAGGTA
GTGGTTTGACAGTTAATAGCTATGAGGAGTTAGAGAGTTTATTTAAAAATAAGGAATTGCATCCAATGGATTTAAAA
AATGCTGTAGCTGAAGAACTTATAAAGATTTTAGAGCCAATTAGAAAGAGATTATAAGTCGACCATCATCATCATCA
TCATTGAGTTTAAACGGTCTCCAGCTTGGCTGTTTTGGCGGATGAGAGAAGATTTTCAGCCTGATACAGATTAAATC
AGAACGCAGAAGCGGTCTGATAAAACAGAATTTGCCTGGCGGCAGTAGCGCGGTGGTCCCACCTGACCCCATGCC

*GAACTCAGAAGTGAAACGCCGTAGCGCCGATGGTAGTGTGGGGTCTCCCCATGCGAGAGTAGGGAACTGCCAGGC
ATCAAATAAAACGAAAGGCTCAGTCGAAAGACTGGGCCTTGTTTGTGAGCTCCCGGTCATCACTGCAGTTTCAAAC
GCTAAATTGCCTGATGCGCTACGCTTATCAGGCCTACATGATCTCTGCAATATATTGAGTTTGCGTGCTTTTGTAGGC
CGGATAAGGCGTTCACGCCGCATCCGGCAAGAAACAGCAAACAATCCAAAACGCCGCGTTCAGCGGCGTTTTTTCT
GCTTTTCTTCGCGAATTAATTCCGCTTCGCAACATGTGAGCACCGGTTTATTGACTACCGGAAGCAGTGTGACCGCA
GCTCAGGGTCGAATTTGCTTTCGAATTTCTGCCATTCATCCGCTTATTATCACTTATTCAGGCGTAGCACCAGGCGTT
TAAGGGCACCAATAACTGCCTTAAAAAAATTACGCCCCGCCCTGCCACTCATCGCAGTACTGTTGTAATTCATTAAG
CATTCTGCCGACATGGAAGCCATCACAGACGGCATGATGAACCTGAATCGCCAGCGGCATCAGCACCTTGTCGCCT
TGCGTATAATATTTGCCCATGGTGAAAACGGGGGCGAAGAAGTTGTCCATATTGGCCACGTTTAAATCAAAACTGGT
GAAACTCACCCAGGGATTGGCTGAGACGAAAAACATATTCTCAATAAACCCTTTAGGGAAATAGGCCAGGTTTTCA
CCGTAACACGCCACATCTTGCGAATATATGTGTAGAAACTGCCGGAAATCGTCGTGGTATTCACTCCAGAGCGATGA
AAACGTTTCAGTTTGCTCATGGAAAACGGTGTAACAAGGGTGAACACTATCCCATATCACCAGCTCACCGTCTTTCA
TTGCCATACGGAATTCCGGATGAGCATTCATCAGGCGGGCAAGAATGTGAATAAAGGCCGGATAAAACTTGTGCTT
ATTTTTCTTTACGGTCTTTAAAAAGGCCGTAATATCCAGCTGAACGGTCTGGTTATAGGTACATTGAGCAACTGACTG
AAATGCCTCAAAATGTTCTTTACGATGCCATTGGGATATATCAACGGTGGTATATCCAGTGATTTTTTTCTCCATTTTA
GCTTCCTTAGC*

pEvole-MVTyrRS

*TCCTGAAAATCTCGATAACTCAAAAAATACGCCCGGTAGTGATCTTATTTCATTATGGTGAAAGTTGGAACCTCTTAC
GTGCCGATCAACGTCTCATTTTCGCCAAAAGTTGGCCCAGGGCTTCCCGGTATCAACAGGGACACCAGGATTTATTT
ATTCTGCGAAGTGATCTTCCGTCACAGGTATTTATTCGGCGCAAAGTGCGTCGGGTGATGCTGCCAACTTACTGATT
TAGTGTATGATGGTGTTTTTGAGGTGCTCCAGTGGCTTCTGTTTCTATCAGCTGTCCCTCCTGTTCAGCTACTGACGG
GGTGGTGCGTAACGGCAAAAGCACCGCCGGACATCAGCGCTAGCGGAGTGTATACTGGCTTACTATGTTGGCACTG
ATGAGGGTGTCAGTGAAGTGCTTCATGTGGCAGGAGAAAAAAGGCTGCACCGGTGCGTCAGCAGAATATGTGATA
CAGGATATATTCCGCTTCCTCGCTCACTGACTCGCTACGCTCGGTCGTTCGACTGCGGCGAGCGGAAATGGCTTACG
AACGGGGCGGAGATTTCCTGGAAGATGCCAGGAAGATACTTAACAGGGAAGTGAGAGGGCCGCGGCAAAGCCGTT
TTTCCATAGGCTCCGCCCCCCTGACAAGCATCACGAAATCTGACGCTCAAATCAGTGGTGGCGAAACCCGACAGGA
CTATAAAGATACCAGGCGTTTCCCCCTGGCGGCTCCCTCGTGCGCTCTCCTGTTCCTGCCTTTCGGTTTACCGGTGTC
ATTCCGCTGTTATGGCCGCGTTTGTCTCATTCCACGCCTGACACTCAGTTCCGGGTAGGCAGTTCGCTCCAAGCTGG
ACTGTATGCACGAACCCCCCGTTCAGTCCGACCGCTGCGCCTTATCCGGTAACTATCGTCTTGAGTCCAACCCGGAA
AGACATGCAAAAGCACCACTGGCAGCAGCCACTGGTAATTGATTTAGAGGAGTTAGTCTTGAAGTCATGCGCCGGT
TAAGGCTAAACTGAAAGGACAAGTTTTGGTGACTGCGCTCCTCCAAGCCAGTTACCTCGGTTCAAAGAGTTGGTAG
CTCAGAGAACCTTCGAAAAACCGCCCTGCAAGGCGGTTTTTTCGTTTTCAGAGCAAGAGATTACGCGCAGACCAAA
ACGATCTCAAGAAGATCATCTTATTAATCAGATAAAATATTTCTAGATTTCAGTGCAATTTATCTCTTCAAATGTAGC
ACCTGAAGTCAGCCCCATACGATATAAGTTGTAATTCTCATGTTTGACAGCTTATCATCGATAAGCTTGGTACCCAAT
TATGACAACTTGACGGCTACATCATTCACTTTTTCTTCACAACCGGCACGGAACTCGCTCGGGCTGGCCCCGGTGCA
TTTTTTAAATACCCGCGAGAAATAGAGTTGATCGTCAAAACCAACATTGCGACCGACGGTGGCGATAGGCATCCGG
GTGGTGCTCAAAAGCAGCTTCGCCTGGCTGATACGTTGGTCCTCGCGCCAGCTTAAGACGCTAATCCCTAACTGCTG
GCGGAAAAGATGTGACAGACGCGACGGCGACAAGCAAACATGCTGTGCGACGCTGGCGATATCAAAATTGCTGTC
TGCCAGGTGATCGCTGATGTACTGACAAGCCTCGCGTACCCGATTATCCATCGGTGGATGGAGCGACTCGTTAATCG
CTTCCATGCGCCGCAGTAACAATTGCTCAAGCAGATTTATCGCCAGCAGCTCCGAATAGCGCCCTTCCCCTTGCCCG
GCGTTAATGATTTGCCCAAACAGGTCGCTGAAATGCGGCTGGTGCGCTTCATCCGGGCGAAAGAACCCCGTATTGG
CAAATATTGACGGCCAGTTAAGCCATTCATGCCAGTAGGCGCGCGGACGAAAGTAAACCCACTGGTGATACCATTC
GCGAGCCTCCGGATGACGACCGTAGTGATGAATCTCTCCTGGCGGGAACAGCAAAATATCACTCGGTCGGCAAACA
AATTCTCGTCCCTGATTTTTCACCACCCCCTGACCGCGAATGGTGAGATTGAGAATATAACCTTTCATTCCCAGCGG*

TCGGTCGATAAAAAAATCGAGATAACCGTTGGCCTCAATCGGCGTTAAACCCGCCACCAGATGGGCATTAAACGAG
TATCCCGGCAGCAGGGGATCATTTTGCGCTTCAGCCATACTTTTCATACTCCCGCCATTCAGAGAAGAAACCAATTG
TCCATATTGCATCAGACATTGCCGTCACTGCGTCTTTTACTGGCTCTTCTCGCTAACCAAACCGGTAACCCCGCTTAT
TAAAAGCATTCTGTAACAAAGCGGGACCAAAGCCATGACAAAAACGCGTAACAAAAGTGTCTATAATCACGGCAG
AAAAGTCCACATTGATTATTTGCACGGCGTCACACTTTGCTATGCCATAGCATTTTTATCCATAAGATTAGCGGATCC
TACCTGACGCTTTTTATCGCAACTCTCTACTGTTTCTCCATACCCGTTTTTTTGGGCTAACAGGAGGAATTAGATCTAT
GGAAAATACCGATCATACCAATAATGAACATCGTCTGACCCAGCTTCTTAGCATAGCGGAAGAATGCGAAACCCTG
GATCGTTTAAAACAGCTGGTGGATTCGGGTCGTATCTTTACTGCGTATAATGGCTTTGAACCGAGCGGCCGTATTCAT
ATTGCCCAGGCGTTGATAACAGTCATGAACACCAACAACATAATTGAATGCGGTGGCCAGATGATTATCTACATCGC
GGACTGGTTTGCGAAAATGAACCTGAAGATGAATGGCGATATTAACAAGATTCGTGAACTGGGCCGCTATTTCATA
GAAGTGTTTAAAGCATGTGGCATAAATCTGGACGGGACCCGTTTCATTTGGGCGAGCGAGTTTATAGCGAGCAATC
CGAGCTACATTGAACGTATGCTGGATATTGCAGAATTTAGCACCATTAGTCGTGTGAAACGTTGCTGCCAGATTATG
GGCCGGAATGAAAGTGACTGCCTGAAAGCGAGCCAAATCTTTTATCCGTGTATGCAAGCGGCGGACGTTTTTGAGT
TAGTACCGGAAGGAATTGATATTTGCCAACTGGGTATTGACCAGCGTAAAGTGAATATGTTGGCGATTGAGTATGCG
AATGACCGTGGTCTGAAAATTCCTATTAGTTTGTCCCATCACATGCTGATGAGCCTGAGCGGTCCGAAGAAAAAGA
TGTCAAAAAGCGATCCGCAGGGCGCGATCTTTATGGATGATACCGAACAGGAAGTGAGCGAAAAGATAAGCCGTG
CGTATTGCACCGATGAAACCTTTGATAATCCGATTTTTGAATATATCAAGTATCTGCTGCTGCGCTGGTTCGGAACCC
TGAATCTGTGCGGCAAAATCTACACCGATATAGAAAGCATTCAGGAAGATTTTAGCAGCATGAATAAACGTGAGCT
GAAGACCGACGTGGCGAACTACATAAACACAATTATCGATCTGGTGCGTGAACATTTCAAGAAACCGGAACTGAGC
GAGCTGCTGTCGAACGTTAAAAGCTATCAGCAGCCGAGCAAATAAGTCGACCATCATCATCATCATCATTGAGTTTA
AACGGTCTCCAGCTTGGCTGTTTTGGCGGATGAGAGAAGATTTTCAGCCTGATACAGATTAAATCAGAACGCAGAA
GCGGTCTGATAAAACAGAATTTGCCTGGCGGCAGTAGCGCGGTGGTCCCACCTGACCCCATGCCGAACTCAGAAGT
GAAACGCCGTAGCGCCGATGGTAGTGTGGGGTCTCCCCATGCGAGAGTAGGGAACTGCCAGGCATCAAATAAAAC
GAAAGGCTCAGTCGAAAGACTGGGCCTTGTTTGTGAGCTCCCGGTCATCACTGCAGTTTCAAACGCTAAATTGCCT
GATGCGCTACGCTTATCAGGCCTACATGATCTCTGCAATATATTGAGTTTGCGTGCTTTTGTAGGCCGGATAAGGCGT
TCACGCCGCATCCGGCAAGAAACAGCAAACAATCCAAAACGCCGCGTTCAGCGGCGTTTTTTCTGCTTTTCTTCGC
GAATTAATTCCGCTTCGCAACATGTGAGCACCGGTTTATTGACTACCGGAAGCAGTGTGACCGCAGCTCAGGGTCG
AATTTGCTTTCGAATTTCTGCCATTCATCCGCTTATTATCACTTATTCAGGCGTAGCACCAGGCGTTTAAGGGCACCA
ATAACTGCCTTAAAAAAATTACGCCCCGCCCTGCCACTCATCGCAGTACTGTTGTAATTCATTAAGCATTCTGCCGAC
ATGGAAGCCATCACAGACGGCATGATGAACCTGAATCGCCAGCGGCATCAGCACCTTGTCGCCTTGCGTATAATATT
TGCCCATGGTGAAAACGGGGGCGAAGAAGTTGTCCATATTGGCCACGTTTAAATCAAAACTGGTGAAACTCACCCA
GGGATTGGCTGAGACGAAAAACATATTCTCAATAAACCCTTTAGGGAAATAGGCCAGGTTTTCACCGTAACACGCC
ACATCTTGCGAATATATGTGTAGAAACTGCCGGAAATCGTCGTGGTATTCACTCCAGAGCGATGAAAACGTTTCAGT
TTGCTCATGGAAAACGGTGTAACAAGGGTGAACACTATCCCATATCACCAGCTCACCGTCTTTCATTGCCATACGGA
ATTCCGGATGAGCATTCATCAGGCGGGCAAGAATGTGAATAAAGGCCGGATAAAACTTGTGCTTATTTTTCTTTACG
GTCTTTAAAAAGGCCGTAATATCCAGCTGAACGGTCTGGTTATAGGTACATTGAGCAACTGACTGAAATGCCTCAAA
ATGTTCTTTACGATGCCATTGGGATATATCAACGGTGGTATATCCAGTGATTTTTTTCTCCATTTTAGCTTCCTTAGC

pBAD-eGFP150TAG-MjtRNA

AAGAAACCAATTGTCCATATTGCATCAGACATTGCCGTCACTGCGTCTTTTACTGGCTCTTCTCGCTAACCAAACCG
GTAACCCCGCTTATTAAAAGCATTCTGTAACAAAGCGGGACCAAAGCCATGACAAAAACGCGTAACAAAAGTGTCT
ATAATCACGGCAGAAAAGTCCACATTGATTATTTGCACGGCGTCACACTTTGCTATGCCATAGCATTTTTATCCATAA
GATTAGCGGATCCTACCTGACGCTTTTTATCGCAACTCTCTACTGTTTCTCCATACCCGTTTTTTTGGGCTAACAGGAG
GAATTAACCATGGTGAGCAAAGGCGAAGAACTGTTCACCGGTGTGGTGCCGATTCTGGTTGAACTGGACGGCGAT
GTGAACGGCCACAAATTCAGCGTGAGTGGCGAAGGTGAAGGCGATGCGACCTACGGCAAACTGACGCTGAAGTTC

ATCTGCACCACGGGCAAACTGCCGGTTCCATGGCCGACGCTGGTGACCACGCTGACCTATGGCGTGCAATGCTTCA
GTCGCTACCCGGACCACATGAAGCAGCACGACTTCTTCAAAAGCGCGATGCCAGAAGGCTACGTGCAAGAACGCA
CCATCTTCTTCAAGGACGACGGCAACTACAAAACCCGCGCCGAGGTGAAATTCGAGGGTGATACGCTGGTGAACC
GCATCGAGCTCAAGGGCATCGACTTCAAAGAAGACGGCAACATTCTGGGCCACAAGCTGGAGTATAACTACAACA
GCCACTAGGTGTACATCATGGCGGACAAGCAGAAAAACGGCATCAAAGTTAACTTCAAGATCCGCCACAACATCGA
GGATGGCAGCGTGCAGCTGGCCGATCATTACCAGCAGAATACGCCGATTGGCGACGGTCCGGTTCTGCTGCCAGAT
AACCACTATCTGAGCACGCAGAGCGCGCTGAGCAAAGATCCGAACGAGAAGCGCGATCACATGGTTCTGCTGGAA
TTCGTGACCGCCGCGGGCATCACCCTCGGTATGGATGAACTGTACAAACATCATCATCATCATCATTAAAAGCTTGG
CTGTTTTGGCGGATGAGAGAAGATTTTCAGCCTGATACAGATTAAATCAGAACGCAGAAGCGGTCTGATAAAACAG
AATTTGCCTGGCGGCAGTAGCGCGGTGGTCCCACCTGACCCCATGCCGAACTCAGAAGTGAAACGCCGTAGCGCCG
ATGGTAGTGTGGGGTCTCCCCATGCGAGAGTAGGGAACTGCCAGGCATCAAATAAAACGAAAGGCTCAGTCGAAA
GACTGGGCCTTTCGTTTTATCTGTTGTTTGTCGGTGAACGCTCTCCTGAGTAGGACAAATCCGCCGGGAGCGGATTT
GAACGTTGCGAAGCAACGGCCCGGAGGGTGGCGGGCAGGACGCCCGCCATAAACTGCCAGGCATCACTGCAGCTG
CTTTTCTTCGCGAATTAATTCCGCTTCGCAACATGTGAGCACCGGTTTATTGACTACCGGAAGCAGTGTGACCGTGT
GCTTCTCAAATGCCTGAGGCCAGTTTGCTCAGGCTCTCCCCGTGGAGGTAATAATTGACGATATGATCAGTGCACGG
CTAACTAAGCGGCCTGCTGACTTTCTCGCCGATCAAAAGGCATTTTGCTATTAAGGGATTGACGAGGGCGTATCTGC
GCAGTAAGATGCGCCCCGCATTCCGGCGGTAGTTCAGCAGGGCAGAACGGCGGACTCTAAATCCGCATGGCAGGG
GTTCAAATCCCCTCCGCCGGACCAAATTCGAAAAGCCTGCTCAACGAGCAGGCTTTTTTGCATGCTCGAGAAGCAG
AAGGCCATCCTGACGGATGGCCTTTTTGCGTTTCTACAAACTCTTTTGTTTATTTTTCTAAATACATTCAAATATGTAT
CCGCTCATGAGACAATAACCCTGATAAATGCTTCAATAATATTGAAAAAGGAAGAGTATGAGTATTCAACATTTCCG
TGTCGCCCTTATTCCCTTTTTTGCGGCATTTTGCCTTCCTGTTTTTGCTCACCCAGAAACGCTGGTGAAAGTAAAAGA
TGCTGAAGATCAGTTGGGTGCACGAGTGGGTTACATCGAACTGGATCTCAACAGCGGTAAGATCCTTGAGAGTTTT
CGCCCCGAAGAACGTTTTCCAATGATGAGCACTTTTAAAGTTCTGCTATGTGGCGCGGTATTATCCCGTGTTGACGC
CGGGCAAGAGCAACTCGGTCGCCGCATACACTATTCTCAGAATGACTTGGTTGAGTACTCACCAGTCACAGAAAAG
CATCTTACGGATGGCATGACAGTAAGAGAATTATGCAGTGCTGCCATAACCATGAGTGATAACACTGCGGCCAACTT
ACTTCTGACAACGATCGGAGGACCGAAGGAGCTAACCGCTTTTTTGCACAACATGGGGGATCATGTAACTCGCCTT
GATCGTTGGGAACCGGAGCTGAATGAAGCCATACCAAACGACGAGCGTGACACCACGATGCCTGTAGCAATGGCA
ACAACGTTGCGCAAACTATTAACTGGCGAACTACTTACTCTAGCTTCCCGGCAACAATTAATAGACTGGATGGAGGC
GGATAAAGTTGCAGGACCACTTCTGCGCTCGGCCCTTCCGGCTGGCTGGTTTATTGCTGATAAATCTGGAGCCGGTG
AGCGTGGGTCTCGCGGTATCATTGCAGCACTGGGGCCAGATGGTAAGCCCTCCCGTATCGTAGTTATCTACACGACG
GGGAGTCAGGCAACTATGGATGAACGAAATAGACAGATCGCTGAGATAGGTGCCTCACTGATTAAGCATTGGTAAC
TGTCAGACCAAGTTTACTCATATATACTTTAGATTGATTTAAAACTTCATTTTTAATTTAAAAGGATCTAGGTGAAGAT
CCTTTTTGATAATCTCATGACCAAAATCCCTTAACGTGAGTTTTCGTTCCACTGAGCGTCAGACCCCGTAGAAAAGA
TCAAAGGATCTTCTTGAGATCCTTTTTTTCTGCGCGTAATCTGCTGCTTGCAAACAAAAAAACCACCGCTACCAGCG
GTGGTTTGTTTGCCGGATCAAGAGCTACCAACTCTTTTTCCGAAGGTAACTGGCTTCAGCAGAGCGCAGATACCAA
ATACTGTCCTTCTAGTGTAGCCGTAGTTAGGCCACCACTTCAAGAACTCTGTAGCACCGCCTACATACCTCGCTCTGC
TAATCCTGTTACCAGTGGCTGCTGCCAGTGGCGATAAGTCGTGTCTTACCGGGTTGGACTCAAGACGATAGTTACCG
GATAAGGCGCAGCGGTCGGGCTGAACGGGGGGTTCGTGCACACAGCCCAGCTTGGAGCGAACGACCTACACCGAA
CTGAGATACCTACAGCGTGAGCTATGAGAAAGCGCCACGCTTCCCGAAGGGAGAAAGGCGGACAGGTATCCGGTA
AGCGGCAGGGTCGGAACAGGAGAGCGCACGAGGGAGCTTCCAGGGGGAAACGCCTGGTATCTTTATAGTCCTGTC
GGGTTTCGCCACCTCTGACTTGAGCGTCGATTTTTGTGATGCTCGTCAGGGGGGCGGAGCCTATGGAAAAACGCCA
GCAACGCGGCCTTTTTACGGTTCCTGGCCTTTTGCTGGCCTTTTGCTCACATGTTCTTTCCTGCGTTATCCCCTGATTC
TGTGGATAACCGTATTACCGCCTTTGAGTGAGCTGATACCGCTCGCCGCAGCCGAACGACCGAGCGCAGCGAGTCA
GTGAGCGAGGAAGCGGAAGAGCGCCTGATGCGGTATTTTCTCCTTACGCATCTGTGCGGTATTTCACACCGCATATG

GTGCACTCTCAGTACAATCTGCTCTGATGCCGCATAGTTAAGCCAGTATACACTCCGCTATCGCTACGTGACTGGGTC
ATGGCTGCGCCCCGACACCCGCCAACACCCGCTGACGCGCCCTGACGGGCTTGTCTGCTCCCGGCATCCGCTTACA
GACAAGCTGTGACCGTCTCCGGGAGCTGCATGTGTCAGAGGTTTTCACCGTCATCACCGAAACGCGCGAGGCAGC
AGATCAATTCGCGCGCGAAGGCGAAGCGGCATGCATAATGTGCCTGTCAAATGGACGAAGCAGGGATTCTGCAAA
CCCTATGCTACTCCGTCAAGCCGTCAATTGTCTGATTCGTTACCAATTATGACAACTTGACGGCTACATCATTCACTT
TTTCTTCACAACCGGCACGGAACTCGCTCGGGCTGGCCCCGGTGCATTTTTTAAATACCCGCGAGAAATAGAGTTGA
TCGTCAAAACCAACATTGCGACCGACGGTGGCGATAGGCATCCGGGTGGTGCTCAAAAGCAGCTTCGCCTGGCTGA
TACGTTGGTCCTCGCGCCAGCTTAAGACGCTAATCCCTAACTGCTGGCGGAAAAGATGTGACAGACGCGACGGCGA
CAAGCAAACATGCTGTGCGACGCTGGCGATATCAAAATTGCTGTCTGCCAGGTGATCGCTGATGTACTGACAAGCC
TCGCGTACCCGATTATCCATCGGTGGATGGAGCGACTCGTTAATCGCTTCCATGCGCCGCAGTAACAATTGCTCAAG
CAGATTTATCGCCAGCAGCTCCGAATAGCGCCCTTCCCCTTGCCCGGCGTTAATGATTTGCCCAAACAGGTCGCTGA
AATGCGGCTGGTGCGCTTCATCCGGGCGAAAGAACCCCGTATTGGCAAATATTGACGGCCAGTTAAGCCATTCATG
CCAGTAGGCGCGCGGACGAAAGTAAACCCACTGGTGATACCATTCGCGAGCCTCCGGATGACGACCGTAGTGATGA
ATCTCTCCTGGCGGGAACAGCAAAATATCACCCGGTCGGCAAACAAATTCTCGTCCCTGATTTTTCACCACCCCCTG
ACCGCGAATGGTGAGATTGAGAATATAACCTTTCATTCCCAGCGGTCGGTCGATAAAAAAATCGAGATAACCGTTG
GCCTCAATCGGCGTTAAACCCGCCACCAGATGGGCATTAAACGAGTATCCCGGCAGCAGGGGATCATTTTGCGCTT
CAGCCATACTTTTCATACTCCCGCCATTCAGAG

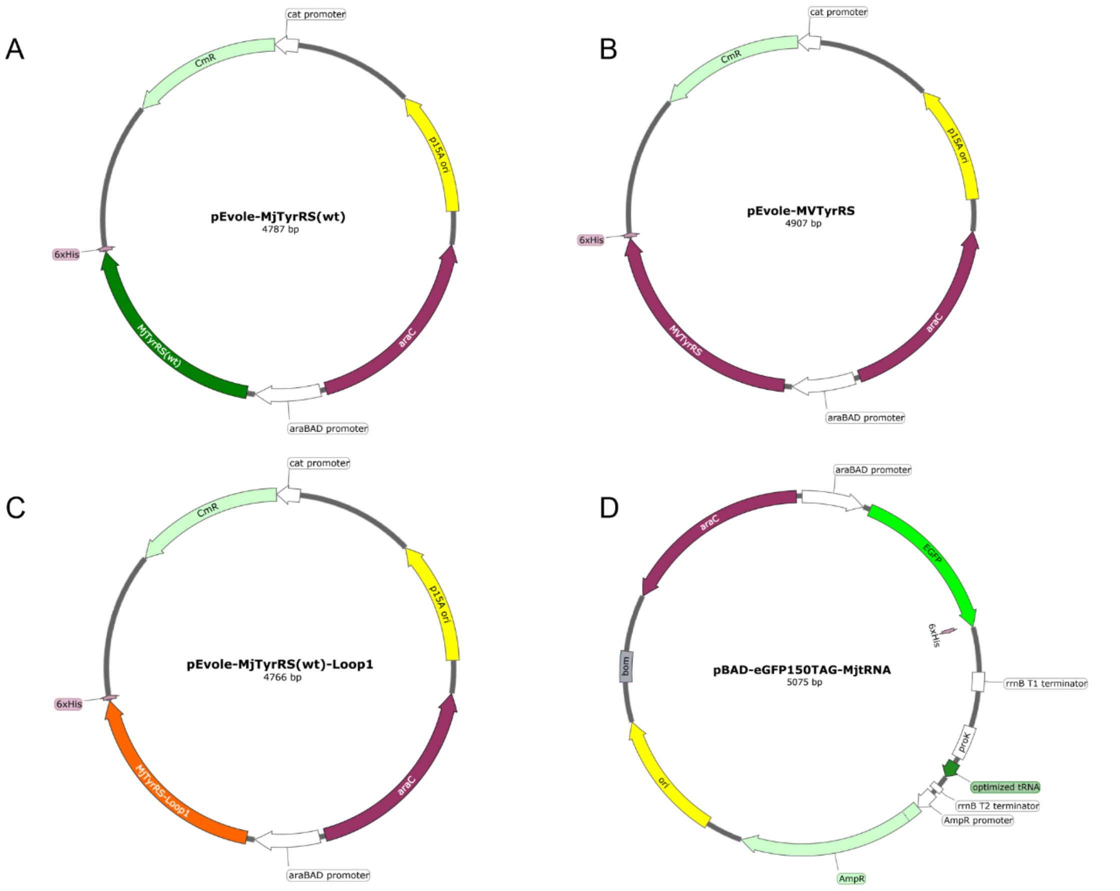

Figure S1. The maps of some plasmids used in this work.(A) pEvole-MjTyrRS(wt) (B) pEvole-MVTyrRS (C) pEvole-MjTyrRS(wt)-Loop1 (D) pBAD-eGFP150TAG-MjtRNA.

```
Methanococcus

Methanococcus     ............................................................
Megavirus         ...MNQFKELLTNVENISLIYNDSDNRVMFTMKSHLLYFLTRFKNSDIVKIIEKDNLKYF
Cafeteria         ...MNQFKELLTNVENISLIYNDSDNRVMFTMKSHLLYFLTRFKNSDIVKIIEKDNLKYF
Megavirus         ...MNQFKELLTNVENISLIYNDSDNHVMFTMKSHLSYFLTRFKNSDVVKIIEKDNLKYF
Megavirus         ...MNQFKELLTNVENISLIYNDSDNHVMFTMKSHLSYFLTRFKNSDVVKIIEKDNLKYF
Mimivirus         ...MNQFKELLTNVKNISLIYNDNDHHVMFTMKSHLSYFLTRFKNSDVVKIIEKDNLEYF
Moumouvirus       ............................................................
Megavirus         ............................................................
Saudi             ............................................................
Cotonvirus        MDNFTRFCAELSQNRDINVINISENEIIFEFVDKSLYFTNKFEEDRIIDITFIDNQKYLC
Acanthamoeba      ............................................................
Acanthamoeba      ............................................................
Mimivirus         ............................................................
Niemeyer          ............................................................
Samba             ............................................................
Mimivirus         ............................................................
Hirudovirus       ............................................................
Acanthamoeba      ............................................................
Acanthamoeba      ............................................................
Niemeyer          ............................................................
Catovirus         ............................................................
Terrestrivirus    ............................................................
Harvfovirus       ............................................................
Orpheovirus       ............................................................
Pandoravirus      ........................................MDDARDQILQDDKNRAFGAS
Pandoravirus      ................................................MMDDTDHQHREK
Pandoravirus      ............................................................
Pandoravirus      ............................................................
Mimivirus         ............................................................
Tupanvirus        ............................................................
Tupanvirus        ............................................................
Marseillevirus    ............................................................
Marseillevirus    ............................................................
Edafosvirus       ............................................................
Hokovirus         ............................................................
Homavirus         ............................................................
Klosneuvirus      ............................................................
Fadolivirus       ............................................................
Megavirus         ...MNQFKELLTNVENISLIYNDSDNHVMFTMKSHLSYFLTRFKNSDVVKIIEKDNLKYF
Megavirus         ...MNQFKELLTNVENISLIYNDSDNHVMFTMKSHLSYFLTRFKNSDVVKIIEKDNLKYF
Acanthamoeba      ............................................................


                                                      α1          β1     α2
Methanococcus                                                                
                                                  1        10          20
Methanococcus     ................................MDEFEMIKRNT..SEIISEEE..LREVL
Megavirus         MLDEISSDNYLDIYDKFIKEYQIYDLDYDNFIDQRVQNLLSIS..EECDTIDR..LKELV
Cafeteria         MLDEISSDNYLDIYDKFIKEYQIYDLDYDNFIDQRVQNLLSIS..EECDTIDR..LKELV
Megavirus         MLDEIASDNYLDVYDKFMKEYQIYDLDYDNFIDQRVQNLLSIS..EECDTIDR..LKELV
Megavirus         MLDEIASDNYLDVYDKFIKEYHIYDLDYDNFIDQRVQNLLSIS..EECDTIDR..LKELV
Mimivirus         IFDEITSDNYLDIYDKFIKQYQIYDLDYDNFIDQRVQNLLSIS..EECDTIDR..LKELV
Moumouvirus       ........................MEQQTLSVEEKFTRLISIS..EECETPDR..LVELI
Megavirus         ........................MEQQILSTEEKITRLISIC..EECETPDR..LEELI
Saudi             ........................MEQQILSTEEKITRLISIC..EECETPDR..LEELI
Cotonvirus        VTREDVDSNFIPDSTYVIKCHDSYVETFDVDVKKRVDQLVAIS..EECDDRDR..LRDLI
Acanthamoeba      .......................MENTDHTNNEHRLTQLLSIA..EECETLDR..LKQLV
Acanthamoeba      .......................MENTDHTNNEHRLTQLLSIA..EECETLDR..LKQLV
Mimivirus         .......................MENTDHTNNEHRLTQLLSIA..EECETLDR..LKQLV
Niemeyer          .......................MENTDHTNNEHRLTQLLSIA..EECETLDR..LKQLV
Samba             .......................MENTDHTNNEHRLTQLLSIA..EECETLDR..LKQLV
Mimivirus         .......................MENTDYTNNEHRLNQLLSIA..EECETLDR..LKQLV
Hirudovirus       .......................MENTDYTNNEHRLNQLLSIA..EECETLDR..LKQLV
Acanthamoeba      .......................MENTDYTNNEHRLNQLLSIA..EECETLDR..LKQLV
Acanthamoeba      .......................MENTDYTNNEHRLNQLLSIA..EECETLDR..LKQLV
Niemeyer          .......................MENTDYSNNEHRLNQLLLIA..EECETLDR..LKQLV
Catovirus         .............................MNIQENIKTIKSIG..EEIIGGDS..LERLL
Terrestrivirus    .............................MNPQEQYDLISHRISPDDIVGKSYL.MEKLQ
Harvfovirus       ...............................MQSRYDSITAGV..KEVINAKY..LKALL
Orpheovirus       ................................MNTYNLITRNL..QEVVGNDEL.LKKID
Pandoravirus      SHTSSSTSTSIQQPAGIIGLPRSRDNIADDDVEYRYGLMTRDL..EEVIGDPAE.IKAVM
Pandoravirus      TEKSVHSQGGNGSPTPIGVDSTARQQTDGDDVEHRYGLVVRDL..DEVIGDPAD.IKAIM
Pandoravirus      .....MIDDDDDGGKETAHLPTDDGALDQDDVEQRYALVVRDL..DEVIGEPAE.IKAIM
Pandoravirus      ..MISDDGDDNSGGKEAAHLPVGNIDPDQGDVEQRYALVVRDL..DEVIGDPAE.IKAIM
Mimivirus         .............................MTELTTFDFITRNL..EPLVGNDAVRLLEII
Tupanvirus        ............................MEVVQDKISLITSRL..QEVIGIDE..LQNII
Tupanvirus        ............................MEIPDDKLSLITSRL..QEIIGLDE..LQKII
Marseillevirus    ................................MSNYDLIVKDL..HEIVGEDI..LKKIA
Marseillevirus    ................................MSNFDLIVKGL..HEIVGKDL..LEKIA
Edafosvirus       ..............................MSEDKYNIITRNL..QEIIGSDD..LKELL
Hokovirus         ..........................MNKLTIDEKRELITKNL..QEILGKQE..LDNLL
Homavirus         ..........................MTNLSVDEKYNLITRNL..QEVLGSEAD.IKSIL
Klosneuvirus      ...........................MELSVDEKFNLITRNL..QETILDEVI.MKKII
Fadolivirus       ...........................MELTVDQKYELITRNL..QEKIVDEEI.MKKIL
Megavirus         MLDEISSNNYLDIYDKFMKEYQIYDLDYDNFIDQRVQNLLSIS..EECDTIDR..LKELV
Megavirus         MLDEISSDNYLDIYDKFIKEYQIYDLDYDNFIDQRVQNLLSIS..EECDTIDR..LKELV
Acanthamoeba      ........................MEQQILSTEEKITRLISIC..EECETPDR..LEELI
```

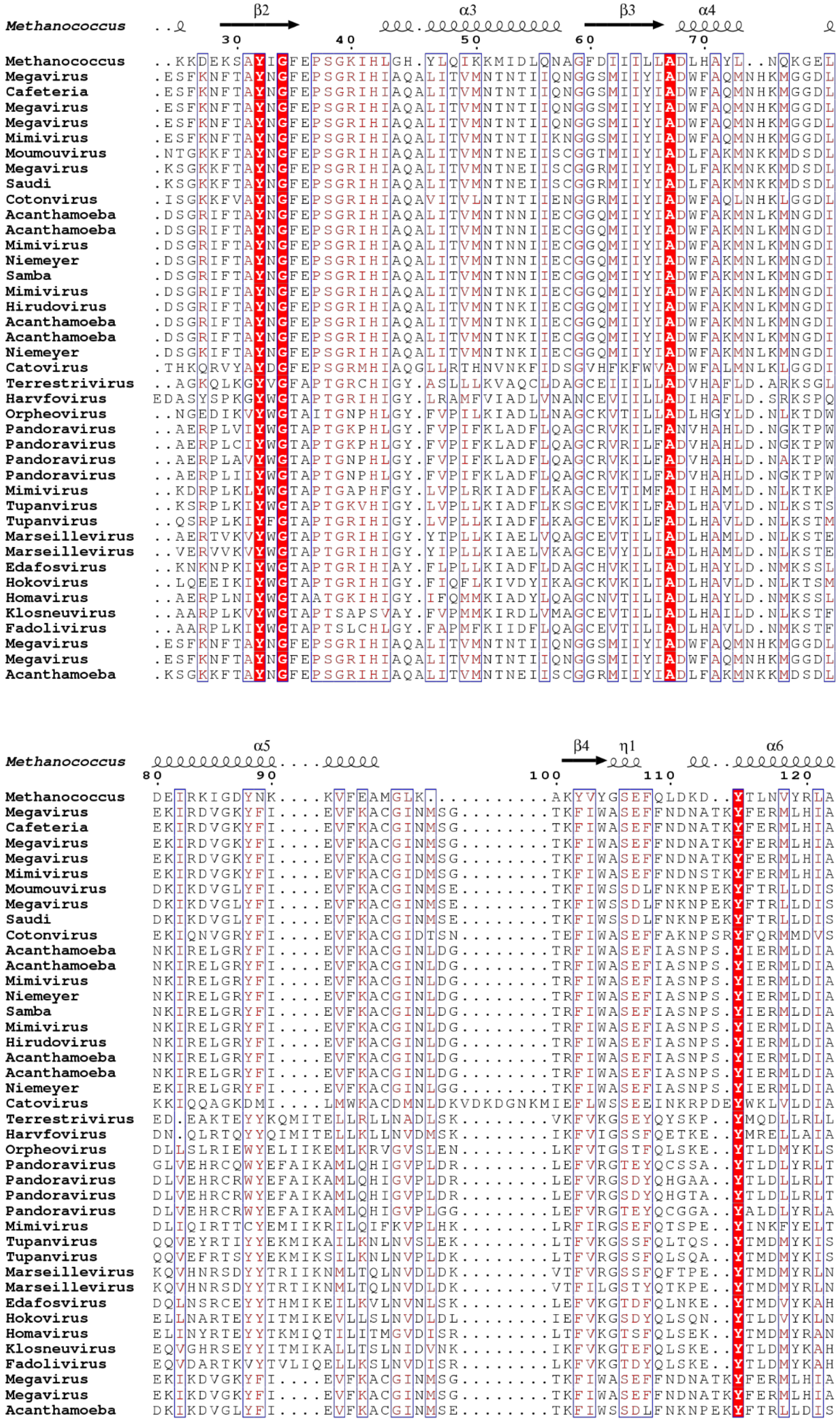
Methanococcus
β2
α3
β3
α4
30
40
50
60
70
Methanococcus
Megavirus
Cafeteria
Megavirus
Megavirus
Mimivirus
Moumouvirus
Megavirus
Saudi
Cotonvirus
Acanthamoeba
Acanthamoeba
Mimivirus
Niemeyer
Samba
Mimivirus
Hirudovirus
Acanthamoeba
Acanthamoeba
Niemeyer
Catovirus
Terrestrivirus
Harvfovirus
Orpheovirus
Pandoravirus
Pandoravirus
Pandoravirus
Pandoravirus
Mimivirus
Tupanvirus
Tupanvirus
Marseillevirus
Marseillevirus
Edafosvirus
Hokovirus
Homavirus
Klosneuvirus
Fadolivirus
Megavirus
Megavirus
Acanthamoeba
Methanococcus
α5
β4
η1
α6
80
90
100
110
120

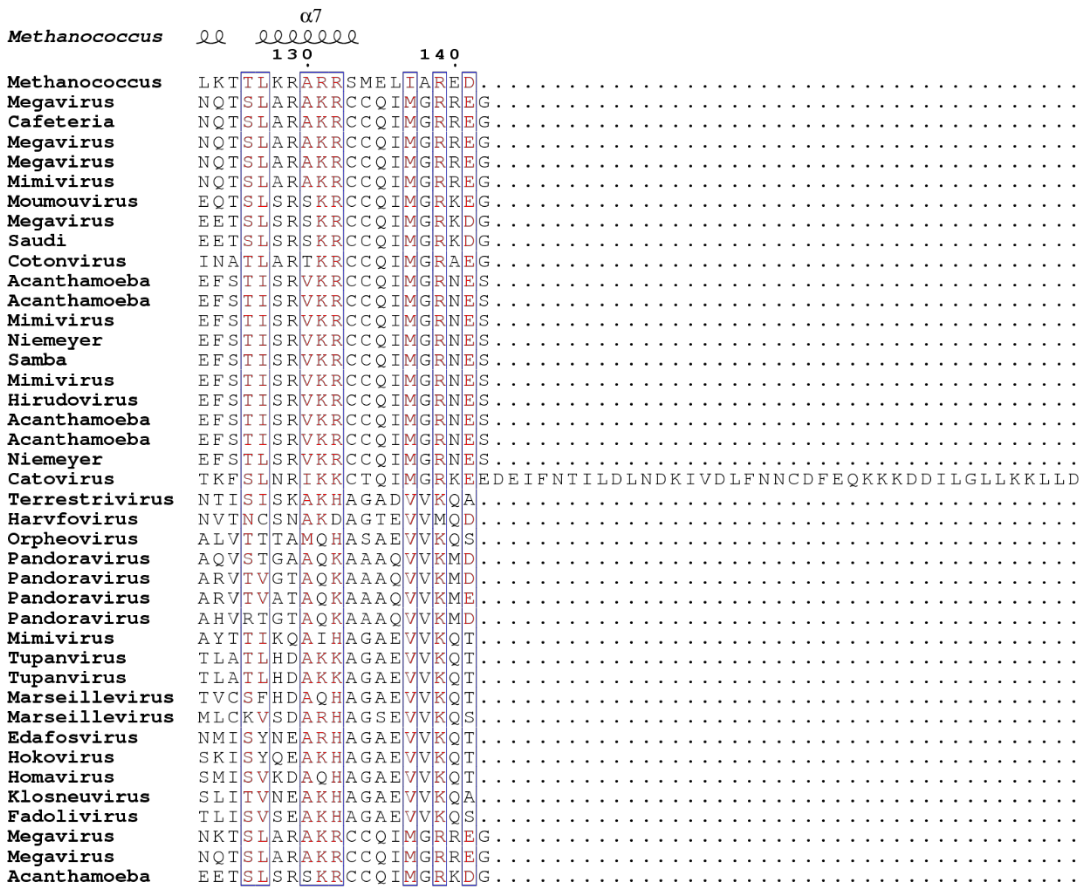

α7
Methanococcus
130
140
Methanococcus LKTTLKRARRSMELIARED...
Megavirus NQTSLARAKRCCQIMGRREG..
Cafeteria NQTSLARAKRCCQIMGRREG..
Megavirus NQTSLARAKRCCQIMGRREG..
Megavirus NQTSLARAKRCCQIMGRREG..
Mimivirus NQTSLARAKRCCQIMGRREG..
Moumouvirus EQTSLSRSKRCCQIMGRKEG..
Megavirus EETSLSRSKRCCQIMGRKDG..
Saudi EETSLSRSKRCCQIMGRKDG..
Cotonvirus INATLARTKRCCQIMGRAEG..
Acanthamoeba EFSTISRVKRCCQIMGRNES..
Acanthamoeba EFSTISRVKRCCQIMGRNES..
Mimivirus EFSTISRVKRCCQIMGRNES..
Niemeyer EFSTISRVKRCCQIMGRNES..
Samba EFSTISRVKRCCQIMGRNES..
Mimivirus EFSTISRVKRCCQIMGRNES..
Hirudovirus EFSTISRVKRCCQIMGRNES..
Acanthamoeba EFSTISRVKRCCQIMGRNES..
Acanthamoeba EFSTISRVKRCCQIMGRNES..
Niemeyer EFSTLSRVKRCCQIMGRNES..
Catovirus TKFSLNRIKKCTQIMGRKEEDEIFNTILDLNDKIVDLFNNCDFEQKKKDDILGLLKKLLD
Terrestrivirus NTISISKAKHAGADVVKQA...
Harvfovirus NVTNCSNAKDAGTEVVMQD...
Orpheovirus ALVTTTAMQHASAEVVKQS...
Pandoravirus AQVSTGAAQKAAAQVVKMD...
Pandoravirus ARVTVGTAQKAAAQVVKMD...
Pandoravirus ARVTVATAQKAAAQVVKME...
Pandoravirus AHVRTGTAQKAAAQVVKMD...
Mimivirus AYTTIKQAIHAGAEVVKQT...
Tupanvirus TLATLHDAKKAGAEVVKQT...
Tupanvirus TLATLHDAKKAGAEVVKQT...
Marseillevirus TVCSFHDAQHAGAEVVKQT...
Marseillevirus MLCKVSDARHAGSEVVKQS...
Edafosvirus NMISYNEARHAGAEVVKQT...
Hokovirus SKISYQEAKHAGAEVVKQT...
Homavirus SMISVKDAQHAGAEVVKQT...
Klosneuvirus SLITVNEAKHAGAEVVKQA...
Fadolivirus TLISVSEAKHAGAEVVKQS...
Megavirus NKTSLARAKRCCQIMGRREG..
Megavirus NQTSLARAKRCCQIMGRREG..
Acanthamoeba EETSLSRSKRCCQIMGRKDG..

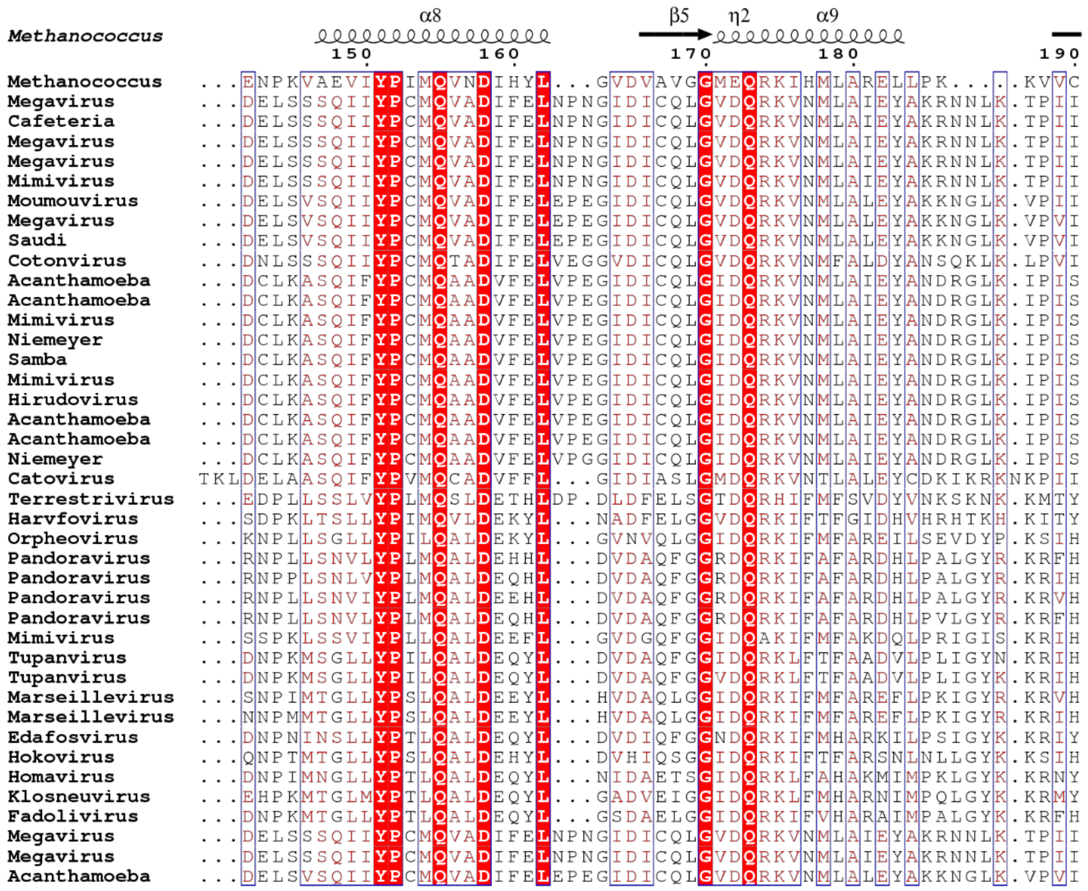

α8
β5
η2
α9
Methanococcus
150
160
170
180
190
Methanococcus ...ENPKVAEVIYPIMQVNDIHYL...GVDVAVGGMEQRKIHMLARELLPK....KVVC
Megavirus ...DELSSSQIIYPCMQVADIFELNPNGIDICQLGVDQRKVNMLAIEYAKRNNLK.TPII
Cafeteria ...DELSSSQIIYPCMQVADIFELNPNGIDICQLGVDQRKVNMLAIEYAKRNNLK.TPII
Megavirus ...DELSSSQIIYPCMQVADIFELNPNGIDICQLGVDQRKVNMLAIEYAKRNNLK.TPII
Megavirus ...DELSSSQIIYPCMQVADIFELNPNGIDICQLGVDQRKVNMLAIEYAKRNNLK.TPII
Mimivirus ...DELSSSQIIYPCMQVADIFELNPNGIDICQLGVDQRKVNMLAIEYAKRNNLK.TPII
Moumouvirus ...DELSVSQIIYPCMQVADIFELEPEGIDICQLGVDQRKVNMLALEYAKKNGLK.VPII
Megavirus ...DELSVSQIIYPCMQVADIFELEPEGIDICQLGVDQRKVNMLALEYAKKNGLK.VPVI
Saudi ...DELSVSQIIYPCMQVADIFELEPEGIDICQLGVDQRKVNMLALEYAKKNGLK.VPVI
Cotonvirus ...DNLSSSQIIYPCMQTADIFELVEGGVDICQLGVDQRKVNMFALDYANSQKLK.LPVI
Acanthamoeba ...DCLKASQIFYPCMQAADVFELVPEGIDICQLGIDQRKVNMLAIEYANDRGLK.IPIS
Acanthamoeba ...DCLKASQIFYPCMQAADVFELVPEGIDICQLGIDQRKVNMLAIEYANDRGLK.IPIS
Mimivirus ...DCLKASQIFYPCMQAADVFELVPEGIDICQLGIDQRKVNMLAIEYANDRGLK.IPIS
Niemeyer ...DCLKASQIFYPCMQAADVFELVPEGIDICQLGIDQRKVNMLAIEYANDRGLK.IPIS
Samba ...DCLKASQIFYPCMQAADVFELVPEGIDICQLGIDQRKVNMLAIEYANDRGLK.IPIS
Mimivirus ...DCLKASQIFYPCMQAADVFELVPEGIDICQLGIDQRKVNMLAIEYANDRGLK.IPIS
Hirudovirus ...DCLKASQIFYPCMQAADVFELVPEGIDICQLGIDQRKVNMLAIEYANDRGLK.IPIS
Acanthamoeba ...DCLKASQIFYPCMQAADVFELVPEGIDICQLGIDQRKVNMLAIEYANDRGLK.IPIS
Acanthamoeba ...DCLKASQIFYPCMQAADVFELVPEGIDICQLGIDQRKVNMLAIEYANDRGLK.IPIS
Niemeyer ...DCLKASQIFYPCMQAADVFELVPGGIDICQLGIDQRKVNMLAIEYANDRGLK.IPIS
Catovirus TKLDELAASQIFYPVMQCADVFFL...GIDIASLGMDQRKVNTLALEYCDKIKRKNKPII
Terrestrivirus ...EDPLLSSLVYPLMQSLDETHLDP.DLDFELSGTDQRHIFMFSVDYVNKSKNK.KMTY
Harvfovirus ...SDPKLTSLLYPIMQVLDEKYL...NADFELGGVDQRKIFTFGIDHVHRHTKH.KITY
Orpheovirus ...KNPLLSGLLYPILQALDEKYL...GVNVQLGGIDQRKIFMFAREILSEVDYP.KSIH
Pandoravirus ...RNPLLSNVLYPLMQALDEHHL...DVDAQFGGRDQRKIFAFARDHLPALGYR.KRFH
Pandoravirus ...RNPPLSNLVYPLMQALDEQHL...DVDAQFGGRDQRKIFAFARDHLPALGYR.KRIH
Pandoravirus ...RNPLLSNVIYPLMQALDEEHL...DVDAQFGGRDQRKIFAFARDHLPALGYR.KRVH
Pandoravirus ...RNPLLSNVLYPLMQALDEQHL...DVDAQFGGRDQRKIFAFARDHLPVLGYR.KRFH
Mimivirus ...SSPKLSSVIYPLLQALDEEFL...GVDGQFGGIDQAKIFMFAKDQLPRIGIS.KRIH
Tupanvirus ...DNPKMSGLLYPILQALDEQYL...DVDAQFGGIDQRKLFTFAADVLPLIGYN.KRIH
Tupanvirus ...DNPKMSGLLYPILQALDEQYL...DVDAQFGGVDQRKLFTFAADVLPLIGYK.KRIH
Marseillevirus ...SNPIMTGLLYPSLQALDEEYL...HVDAQLGGIDQRKIFMFAREFLPKIGYR.KRVH
Marseillevirus ...NNPMMTGLLYPSLQALDEEYL...HVDAQLGGIDQRKIFMFAREFLPKIGYR.KRIH
Edafosvirus ...DNPNINSLLYPTLQALDEQYL...DVDIQFGGNDQRKIFMHARKILPSIGYK.KRIY
Hokovirus ...QNPTMTGLLYPSLQALDEHYL...DVHIQSGGIDQRKIFTFARSNLNLLGYK.KSIH
Homavirus ...DNPIMNGLLYPTLQALDEQYL...NIDAETSGIDQRKLFAHAKMIMPKLGYK.KRNY
Klosneuvirus ...EHPKMTGLMYPTLQALDEQYL...GADVEIGGIDQRKLFMHARNIMPQLGYK.KRMY
Fadolivirus ...DNPKMTGLLYPTLQALDEQYL...GSDAELGGIDQRKIFVHARAIMPALGYK.KRFH
Megavirus ...DELSSSQIIYPCMQVADIFELNPNGIDICQLGVDQRKVNMLAIEYAKRNNLK.TPII
Megavirus ...DELSSSQIIYPCMQVADIFELNPNGIDICQLGVDQRKVNMLAIEYAKRNNLK.TPII
Acanthamoeba ...DELSVSQIIYPCMQVADIFELEPEGIDICQLGVDQRKVNMLALEYAKKNGLK.VPVI

```
                  β6
Methanococcus     ──→      TT
                          200
Methanococcus     IHNPVLTGLDG.................................................
Megavirus         LSHHMLMGLKGS................................................
Cafeteria         LSHHMLMGLKGS................................................
Megavirus         LSHHMLMGLKGS................................................
Megavirus         LSHHMLMGLKGS................................................
Mimivirus         LSHHMLMGLKGS................................................
Moumouvirus       LSHHMLMGLGGP................................................
Megavirus         LSHHMLMGLGGP................................................
Saudi             LSHHMLMGLGGP................................................
Cotonvirus        LSHHMIMGLRGP................................................
Acanthamoeba      LSHHMLMSLSGP................................................
Acanthamoeba      LSHHMLMSLSGP................................................
Mimivirus         LSHHMLMSLSGP................................................
Niemeyer          LSHHMLMSLSGP................................................
Samba             LSHHMLMSLSGP................................................
Mimivirus         LSHHMLMSLSGP................................................
Hirudovirus       LSHHMLMSLSGP................................................
Acanthamoeba      LSHHMLMSLSGP................................................
Acanthamoeba      LSHHMLMSLSGT................................................
Niemeyer          LSHHMLMSLNGP................................................
Catovirus         ISHHMLKGLDG.................................................
Terrestrivirus    IMNQLVPGLSKESLRN.........................................PET
Harvfovirus       LMNSMVPALSGISGDS............................................
Orpheovirus       LMNPLVPGLGKPVKGN.......................................KGKNE
Pandoravirus      LLNPLVPGLTK.................................................
Pandoravirus      LLNPLVPGLTK.................................................
Pandoravirus      LLNPLVPGLTK.................................................
Pandoravirus      LLNPLVPGLTE.................................................
Mimivirus         LMNALIPGLKK.................................................
Tupanvirus        LMNPMLTAINAMPKNI.........................................NED
Tupanvirus        LMNPMLTAINAMPKDS.........................................NEN
Marseillevirus    LLTPMVPGLRCCASTQ............................................
Marseillevirus    LMTPMVPGIRHCASSK............................................
Edafosvirus       LMNSIIPALSKVSLKD........................................PQQE
Hokovirus         LMTPMVQGLRFNKKEI..........KNVITKEEKLKLLNIDFDLPDNYVDTLYKKYINN
Homavirus         FMTEMISGLRFVKKQEQSHSQPVKLNRDDIINLLNIESDDALINNLQEMIDKYNKEKELK
Klosneuvirus      LMNKMISGLRTQKQEP.........................................KEG
Fadolivirus       LMNRMVPGLRFEKTTP..LQSVQNEQKESLKEKVLSLVNSEADEKVLADRLQSLLENNDQ
Megavirus         LSHHMLMGLKGS................................................
Megavirus         LSHHMLMGLKGS................................................
Acanthamoeba      LSHHMLMGLGGP................................................
```

```
                                                 α10                      α11
Methanococcus                        TT    ℓℓℓℓℓℓℓℓℓℓ        TT      ℓℓℓℓℓℓℓℓ
                              210       220       230       240       250
Methanococcus     ....EGKMSSSKGNF.IAVDDSPEEIRAKIKKAYCPAGVV....EGNPIMEIAKYFLEYP
Megavirus         ....KNKMSKSDPENAIFMEDSREDIFKKIHKAYCTDEII.....DNPIYEYIKYIL.LR
Cafeteria         ....KNKMSKSDPENAIFMEDSREDIFKKIHKAYCTDEII.....DNPIYEYIKYIL.LR
Megavirus         ....KNKMSKSDPENAIFMEDSREDIFKKIYKAYCTDEII.....DNPIYEYIKYIL.LR
Megavirus         ....KNKMSKSDPENAIFMEDSREDIFKKIYKAYCTDEII.....DNPIYEYIKYIL.LR
Mimivirus         ....KNKMSKSDPENAIFMEDSREDIFKKIYKAYCTDAIT.....DNPIYEYIKYIL.LR
Moumouvirus       ....KNKMSKSDPKSAIFMEDSYEEIKEKILRAFCTDDTK.....DNPIFEYIKYIL.FR
Megavirus         ....KNKMSKSDPKSAIFMEDSYEEIKEKILRAFCTDDIN.....ENPIFEYIKHIL.FR
Saudi             ....KNKMSKSDPKSAIFMEDSYEEIKEKILRAFCTDDIN.....ENPIFEYIKHIL.FR
Cotonvirus        ....KHKMSKSDPLNAIFMEDTTQEIHDKISKAFCPDTHL.....DNPIFEYIKYIL.LR
Acanthamoeba      ....KKKMSKSDPQGAIFMDDTEQEVSEKISRAYCTDETF.....DNPIFEYIKYLL.LR
Acanthamoeba      ....KKKMSKSDPQGAIFMDDTEQEVSEKISRAYCTDETF.....DNPIFEYIKYLL.LR
Mimivirus         ....KKKMSKSDPQGAIFMDDTEQEVSEKISRAYCTDETF.....DNPIFEYIKYLL.LR
Niemeyer          ....KKKMSKSDPQGAIFMDDTEQEVSEKISRAYCTDETF.....DNPIFEYIKYLL.LR
Samba             ....KKKMSKSDPQGAIFMDDTEQEVSEKISRAYCTDETF.....DNPIFEYIKYLL.LR
Mimivirus         ....KKKMSKSDPQGAIFMDDTEQEVSEKISRAYCTDEIF.....DNPIFEYIKYLL.LR
Hirudovirus       ....KKKMSKSDPQGAIFMDDTEQEVSEKISRAYCTDEIF.....DNPIFEYIKYLL.LR
Acanthamoeba      ....KKKMSKSDPQGAIFMDDTEQEVSEKISRAYCTDEIF.....DNPIFEYIKYLL.LR
Acanthamoeba      ....KKKMSKSDPQGAIFMDDTEQEVSEKISRAYCTDEIF.....DNPIFEYIKYLL.LR
Niemeyer          ....KKKMSKSDPQSAIFMDDTEQEINEKISRAYCTDEIF.....DNPIFEYIKYLL.LR
Catovirus         ....SDKMSKSNPDNTIFMDDSETEIIRKIKKSFCEPGNI....DKNPLLDWIEHLI.LP
Terrestrivirus    KVVTSQKMSSSDSVGKLDLLDTPKEILNKLKKAYCLEGDA....EDNTVLVMCKHII.FP
Harvfovirus       ....GVKMSASGSVGKLDLLDSPKELLKKIKKVYCLQGDI....ADNTLLQLCKHIV.FP
Orpheovirus       GNDIEGKMSSSDKNSKIDFSDSDEVIKDKMKKAYSVDGQV....EGNCLMSMCKLIL.FP
Pandoravirus      ....DGKMSASEAASKIDLDDAEEAIRAKIRRAYSVDGRA....EGNGLLAILRFVL.WR
Pandoravirus      ....DGKMSASEAASKIDLDDCDEVIRAKIRRAYSVDGRA....EGNGLLAILRFIL.WR
Pandoravirus      ....SGKMSASEAASKIGLDDPDEVIRAKIRQAYSVDGCA....EGNGLLAILRFVL.WR
Pandoravirus      ....SGKMSASEAASKIGLDDPDEVIRAKIRRAYSVDGCA....EGNGLLAILRFVL.WR
Mimivirus         ....GGKMSSSIPYSKIDFRDTDEDINRKILGAFSIDGHVGNGKNKNCMLAMMEHVI.YP
Tupanvirus        TNLSEVKMSSSDLNSKIDMMDSKNEVKKKINRAYCLEGDL....SFNPLMELMKLVI.FP
Tupanvirus        TNISEIKMSSSDLNSKIDMLDSKNEIKKKINRAYCLEGDL....SFNPLMELMKHVT.FP
Marseillevirus    ..DNEDKMSTSNTKTKLDMLDSRNEIKKKIAAAYCLPGDI....TDNSVLDILDRVL.FP
Marseillevirus    SMDSQEKMSASDTKTKLDMLDTRNQIKKKIASAFCIPGDV....TDNSILDILERVL.FP
Edafosvirus       STITNKKMSSSDNTSKIDVLEDEKSIKKKINSTYCLEGDI....SDNTLLTMSKLLI.FP
Hokovirus         DTIKDDKMSSSNNDSKIDLLDTKNEIKKKINKSYCLPCDV....EDNCLMDLLEKVI.FP
Homavirus         NNIQLEKMSSSDNNSKIDLLDTKNQIKAKINKCYCLAGDI....KDNCLMNILEKLL.FP
Klosneuvirus      ENIQDQKMSSSNQDSKIDLLDSKNQLKAKINKAYCFPGDI....NDNCLLEMLENLV.FP
Fadolivirus       SNIQLEKMSSSNADSKISLLDTRNQIRSKINKAYCLPGDV....DDNCLLTMLDKIV.FP
Megavirus         ....KNKMSKSDPENAIFMEDSREDIFKKIHKAYCTDEII.....DNPIYEYIKYIL.LR
Megavirus         ....KNKMSKSDPENAIFMEDSREDIFKKIYKAYCTDEIV.....DNPIYEYIKYIL.LR
Acanthamoeba      ....KNKMSKSDPKSAIFMEDSYEEIKEKILRAFCTDDIN.....ENPIFEYIKHIL.FR
```

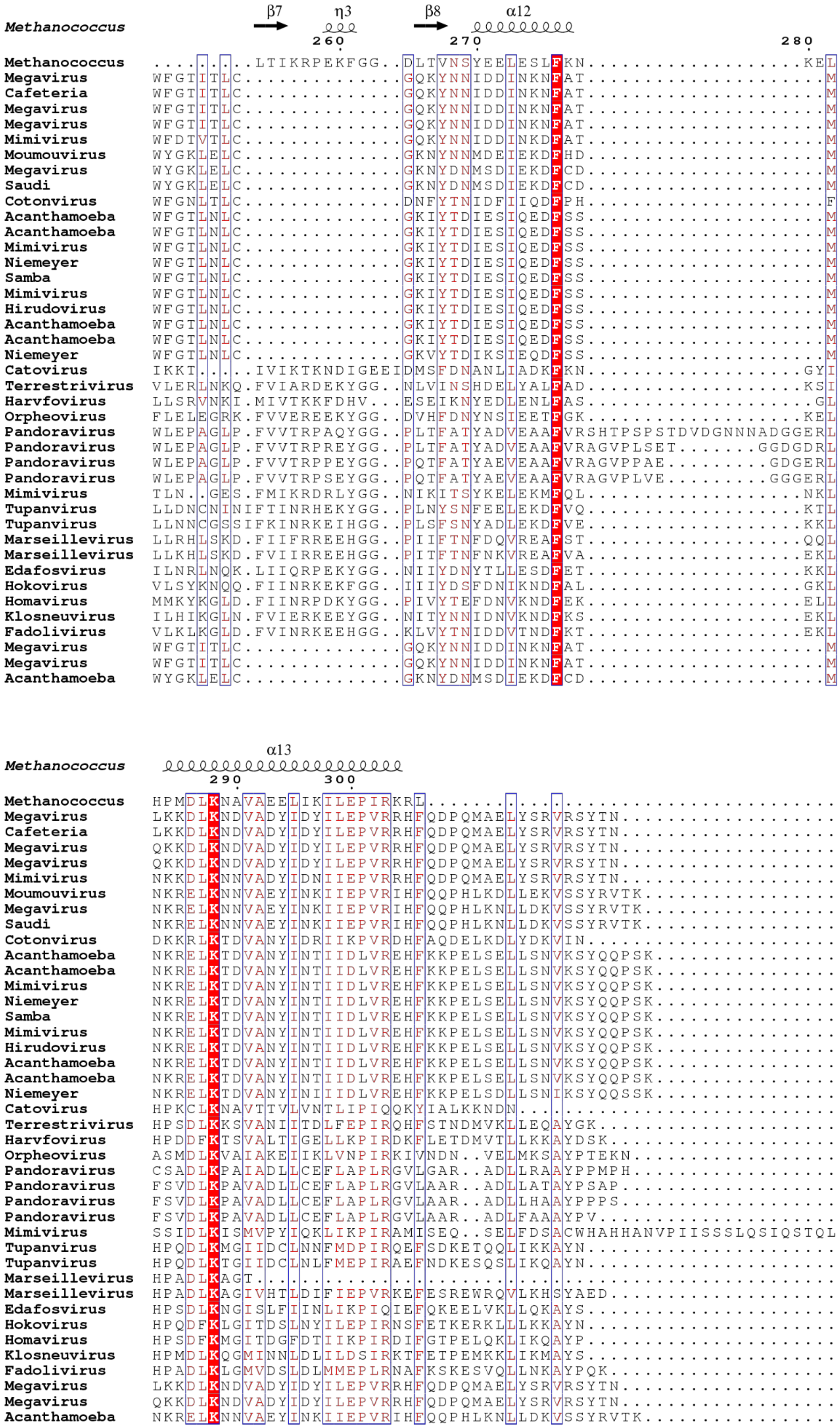

Methanococcus
β7 η3 β8 α12
260 270 280
Methanococcus ......LTIKRPEKFGG..DLTVNSYEELESLFKN..................KEL
Megavirus WFGTITLC.............GQKYNNIDDINKNFAT...................M
Cafeteria WFGTITLC.............GQKYNNIDDINKNFAT...................M
Megavirus WFGTITLC.............GQKYNNIDDINKNFAT...................M
Megavirus WFGTITLC.............GQKYNNIDDINKNFAT...................M
Mimivirus WFDTVTLC.............GQKYNNIDDINKDFAT...................M
Moumouvirus WYGKLELC.............GKNYNNMDEIEKDFHD...................M
Megavirus WYGKLELC.............GKNYDNMSDIEKDFCD...................M
Saudi WYGKLELC.............GKNYDNMSDIEKDFCD...................M
Cotonvirus WFGNLTLC.............DNFYTNIDFIIQDFPH...................F
Acanthamoeba WFGTLNLC.............GKIYTDIESIQEDFSS...................M
Acanthamoeba WFGTLNLC.............GKIYTDIESIQEDFSS...................M
Mimivirus WFGTLNLC.............GKIYTDIESIQEDFSS...................M
Niemeyer WFGTLNLC.............GKIYTDIESIQEDFSS...................M
Samba WFGTLNLC.............GKIYTDIESIQEDFSS...................M
Mimivirus WFGTLNLC.............GKIYTDIESIQEDFSS...................M
Hirudovirus WFGTLNLC.............GKIYTDIESIQEDFSS...................M
Acanthamoeba WFGTLNLC.............GKIYTDIESIQEDFSS...................M
Acanthamoeba WFGTLNLC.............GKIYTDIESIQEDFSS...................M
Niemeyer WFGTLNLC.............GKVYTDIKSIEQDFSS...................M
Catovirus IKKT.....IVIKTKNDIGEEIDMSFDNANLIADKFKN.................GYI
Terrestrivirus VLERLNKQ.FVIARDEKYGG..NLVINSHDELYALFAD.................KSI
Harvfovirus LLSRVNKI.MIVTKKFDHV...ESEIKNYEDLENLFAS..................GL
Orpheovirus FLELEGRK.FVVEREEKYGG..DVHFDNYNSIEETFGK.................KEL
Pandoravirus WLEPAGLP.FVVTRPAQYGG..PLTFATYADVEAAFVRSHTPSPSTDVDGNNNADGGERL
Pandoravirus WLEPAGLP.FVVTRPREYGG..PLTFATYADVEAAFVRAGVPLSET.......GGDGDRL
Pandoravirus WLEPAGLP.FVVTRPPEYGG..PQTFATYAEVEAAFVRAGVPPAE.........GDGERL
Pandoravirus WLEPAGLP.FVVTRPSEYGG..PQTFATYAEVEAAFVRAGVPLVE........GGGERL
Mimivirus TLN..GES.FMIKRDRLYGG..NIKITSYKELEKMFQL..................NKL
Tupanvirus LLDNCNINIFTINRHEKYGG..PLNYSNFEELEKDFVQ..................KTL
Tupanvirus LLNNCGSSIFKINRKEIHGG..PLSFSNYADLEKDFVE..................KKL
Marseillevirus LLRHLSKD.FIIFRREEHGG..PIIFTNFDQVREAFST..................QQL
Marseillevirus LLKHLSKD.FVIIRREEHGG..PITFTNFNKVREAFVA..................EKL
Edafosvirus ILNRLNQK.LIIQRPEKYGG..NIIYDNYTLLESDFET..................KKL
Hokovirus VLSYKNQQ.FIINRKEKFGG..IIIYDSFDNIKNDFAL..................GKL
Homavirus MMKYKGLD.FIINRPDKYGG..PIVYTEFDNVKNDFEK..................ELL
Klosneuvirus ILHIKGLN.FVIERKEEYGG..NITYNNIDNVKNDFKS..................EKL
Fadolivirus VLKLKGLD.FVINRKEEHGG..KLVYTNIDDVTNDFKT..................EKL
Megavirus WFGTITLC.............GQKYNNIDDINKNFAT...................M
Megavirus WFGTITLC.............GQKYNNIDDINKNFAT...................M
Acanthamoeba WYGKLELC.............GKNYDNMSDIEKDFCD...................M
Methanococcus
α13
290 300
Methanococcus HPMDLKNAVAEELIKILEPIRKRL......................................
Megavirus LKKDLKNDVADYIDYILEPVRRHFQDPQMAELYSRVRSYTN...................
Cafeteria LKKDLKNDVADYIDYILEPVRRHFQDPQMAELYSRVRSYTN...................
Megavirus QKKDLKNDVADYIDYILEPVRRHFQDPQMAELYSRVRSYTN...................
Megavirus QKKDLKNDVADYIDYILEPVRRHFQDPQMAELYSRVRSYTN...................
Mimivirus NKKDLKNNVADYIDNIIEPVRRHFQDPQMAELYSRVRSYTN...................
Moumouvirus NKRELKNNVAEYINKIIEPVRIHFQQPHLKDLLEKVSSYRVTK.................
Megavirus NKRELKNNVAEYINKIIEPVRIHFQQPHLKNLLDKVSSYRVTK.................
Saudi NKRELKNNVAEYINKIIEPVRIHFQQPHLKNLLDKVSSYRVTK.................
Cotonvirus DKKRLKTDVANYIDRIIKPVRDHFAQDELKDLYDKVIN......................
Acanthamoeba NKRELKTDVANYINTIIDLVREHFKKPELSELLSNVKSYQQPSK................
Acanthamoeba NKRELKTDVANYINTIIDLVREHFKKPELSELLSNVKSYQQPSK................
Mimivirus NKRELKTDVANYINTIIDLVREHFKKPELSELLSNVKSYQQPSK................
Niemeyer NKRELKTDVANYINTIIDLVREHFKKPELSELLSNVKSYQQPSK................
Samba NKRELKTDVANYINTIIDLVREHFKKPELSELLSNVKSYQQPSK................
Mimivirus NKRELKTDVANYINTIIDLVREHFKKPELSELLSNVKSYQQPSK................
Hirudovirus NKRELKTDVANYINTIIDLVREHFKKPELSELLSNVKSYQQPSK................
Acanthamoeba NKRELKTDVANYINTIIDLVREHFKKPELSELLSNVKSYQQPSK................
Acanthamoeba NKRELKTDVANYINTIIDLVREHFKKPELSELLSNVKSYQQPSK................
Niemeyer NKRELKTDVANYINIIIDLVREHFKKPELSDLLSNIKSYQQSSK................
Catovirus HPKCLKNAVTTVLVNTLIPIQQKYIALKKNDN............................
Terrestrivirus HPSDLKKSVANIITDLFEPIRQHFSTNDMVKLLEQAYGK.....................
Harvfovirus HPDDFKTSVALTIGELLKPIRDKFLETDMVTLLKKAYDSK....................
Orpheovirus ASMDLKVAIAKEIIKLVNPIRKIVNDN..VELMKSAYPTEKN..................
Pandoravirus CSADLKPAIADLLCEFLAPLRGVLGAR..ADLLRAAYPPMPH..................
Pandoravirus FSVDLKPAVADLLCEFLAPLRGVLAAR..ADLLATAYPSAP...................
Pandoravirus FSVDLKPAVADLLCEFLAPLRGVLAAR..ADLLHAAYPPPS...................
Pandoravirus FSVDLKPAVADLLCEFLAPLRGVLAAR..ADLFAAAYPV.....................
Mimivirus SSIDLKISMVPYIQKLIKPIRAMISEQ..SELFDSACWHAHHANVPIISSSLQSIQSTQL
Tupanvirus HPQDLKMGIIDCLNNFMDPIRQEFSDKETQQLIKKAYN......................
Tupanvirus HPQDLKTGIIDCLNLFMEPIRAEFNDKESQSLIKQAYN......................
Marseillevirus HPADLKAGT...
Marseillevirus HPADLKAGIVHTLDIFIEPVRKEFESREWRQVLKHSYAED....................
Edafosvirus HPSDLKNGISLFIINLIKPIQIEFQKEELVKLLQKAYS......................
Hokovirus HPQDFKLGITDSLNYILEPIRNSFETKERKLLLKKAYN......................
Homavirus HPSDFKMGITDGFDTIIKPIRDIFGTPELQKLIKQAYP......................
Klosneuvirus HPMDLKQGMINNLDLILDSIRKTFETPEMKKLIKMAYS......................
Fadolivirus HPADLKLGMVDSLDLMMEPLRNAFKSKESVQLLNKAYPQK....................
Megavirus LKKDLKNDVADYIDYILEPVRRHFQDPQMAELYSRVRSYTN...................
Megavirus QKKDLKNDVADYIDYILEPVRRHFQDPQMAELYSRVRSYTN...................
Acanthamoeba NKRELKNNVAEYINKIIEPVRIHFQQPHLKNLLDKVSSYRVTK.................

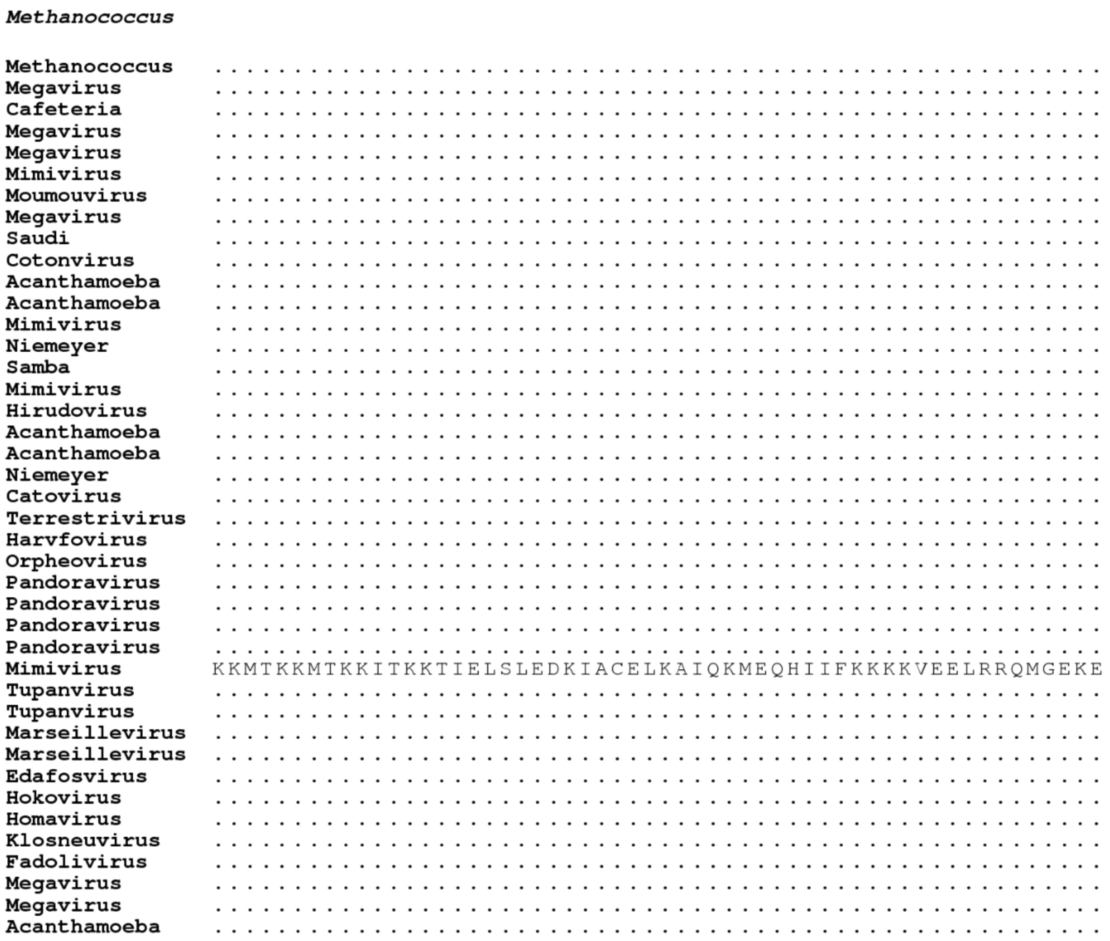

Figure S2. Sequence alignment of MjTyrRS and 40 giant virus derived TyrRSs. The TyrRSs are from *Methanococcus jannaschii*; *Megavirus chiliensis*; *Cafeteria roenbergensis virus BV-PW1*; *Megavirus courdo7-2*; *Megavirus vitis*; *Mimivirus sp. SH*; *Moumouvirus australiensis*; *egavirus courdo 7-1*; *Saudi moumouvirus*; *Cotonvirus japonicus*; *Acanthamoeba castellanii mimivirus*; *Acanthamoeba polyphaga mimivirus*; *Mimivirus Bombay*; *Niemeyer virus-1*; *Samba virus*; *Mimivirus reunion*; *Hirudovirus strain Sangsue*; *Acanthamoeba castellanii mamavirus*; *Acanthamoeba polyphaga lentillevirus*; *Niemeyer virus-2*; *Catovirus CTV1*; *Terrestrivirus sp.*; *Harvfovirus sp.*; *Orpheovirus IHUMI-LCC2*; *Pandoravirus macleodensis*; *Pandoravirus salinus*; *Pandoravirus japonicus*; *Pandoravirus dulcis*; *Mimivirus LCMiAC01*; *Tupanvirus soda lake*; *Tupanvirus deep ocean*; *Marseillevirus LCMAC202*; *Marseillevirus LCMAC201*; *Edafosvirus sp.*; *Hokovirus HKV1*; *Homavirus sp.*; *Klosneuvirus KNV1*; *Fadolivirus 1, Megavirus courdo11,Megavirus lba isolate LBA111,Acanthamoeba polyphaga moumouvirus*. The secondary structural elements of the *M. jannaschii* are indicated above the alignment as coils for α-helices and arrows for β-strands. The residues that are conserved among the *M. jannaschii* and giant viruses are marked in red, and slightly less conservative residues are in red font and framed by blue boxes. Alignments were done with ClustalX2. The figure was drawn with ESPript.

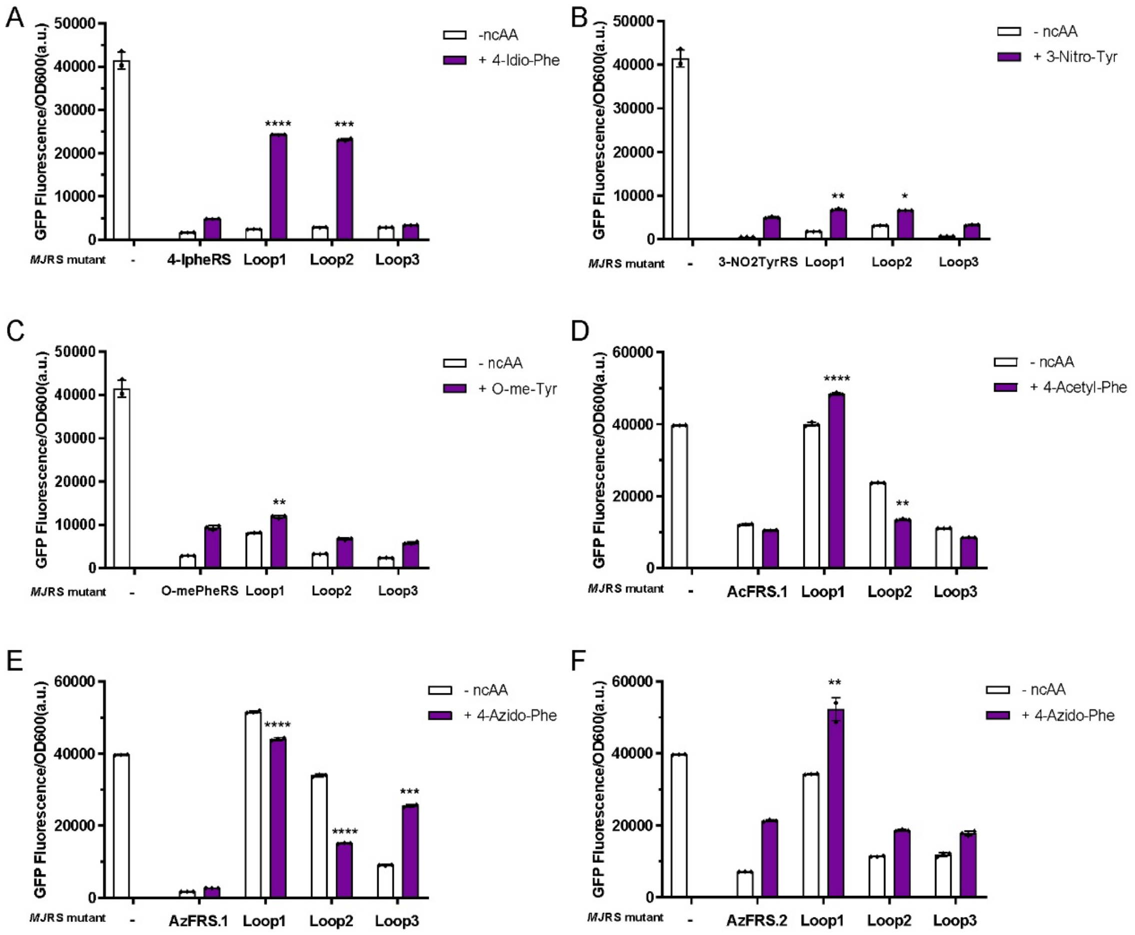

Figure S3. The fluorescence assays of the MjTyrRS loop-minimized mutants to incorporate ncAAs. For the protein expression, pEvolve bears MjTyrRS and pBAD plasmid bears tRNA and GFP reporter. (A) 4-I-Phe MjTyrRS mutant (B)3-NO_2-Tyr MjTyrRS mutant (C) O-Me-Tyr MjTyrRS mutant (D) AcFRS.1 (E)AzFRS.1 (F) AzFRS.2 Error bars represented the ±standard error of the mean from three biologically independent experiments. Statistical significance is quantified with ordinary one-way ANOVA.

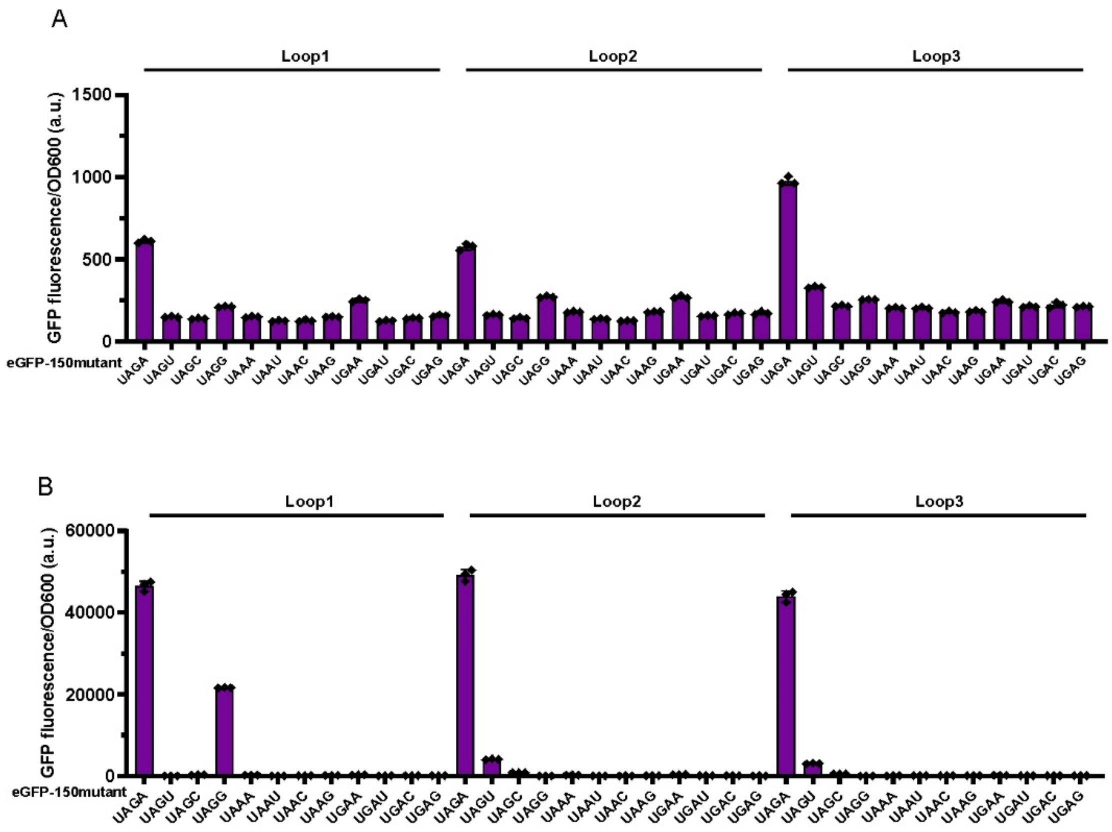

Figure S4. Analysis of quadruplet codons suppression activity of the loop-minimized MjTyrRS. The suppression efficiency is characterized by eGFP reporter assay in *E. coli* (A) DH10B and (B) C321.ΔA.expΔPBAD strain respectively. Error bars represented ± standard error of the mean from three biologically independent experiments.

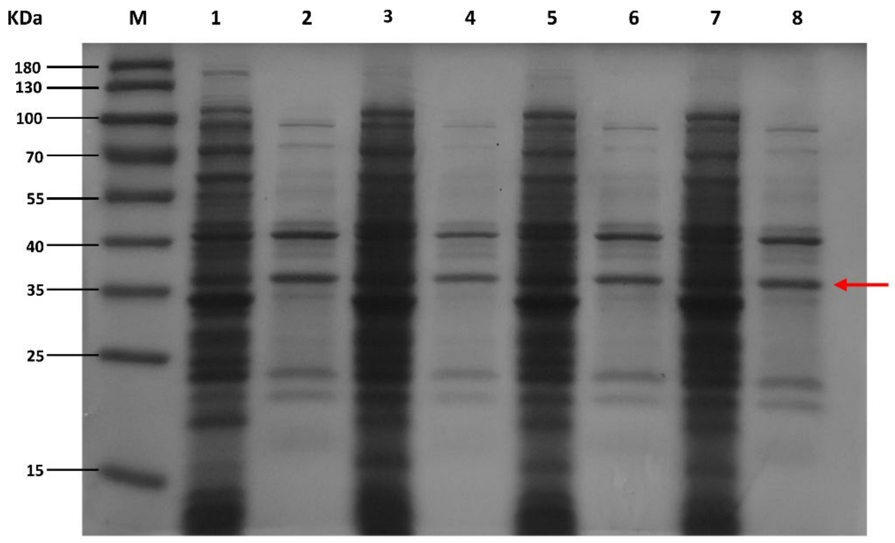

Figure S5. The SDS-PAGE result of the supernatant and precipitate fractions of the MjTyrRS and its loop-minimized mutants. After overnight expression, the bacteria were harvested and lysed by Bugbuster. The supernatant and precipitate fractions were subsequently separated by centrifugation for SDS-PAGE separately. The lanes in the gel are: M: 180 kDa Prestained

Protein Marker; Lane 1: MjTyrRS precipitate fraction; Lane 2: MjTyrRS supernatant fraction; Lane 3: MjTyrRS Loop1 mutant precipitate fraction; Lane 4: MjTyrRS Loop1 mutant supernatant fraction; Lane 5: MjTyrRS Loop2 mutant precipitate fraction; Lane 6: MjTyrRS Loop2 mutant supernatant fraction; Lane 7: MjTyrRS Loop3 mutant precipitate fraction; Lane 8: MjTyrRS Loop3 mutant supernatant fraction. The red arrow points to the position corresponding to the size of MjTyrRS.

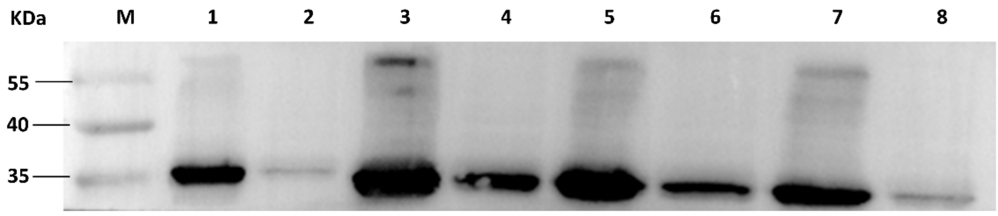

Figure S6. The Western Blot result of the supernatant and precipitate fractions of the MjTyrRS and its loop-minimized mutants. After the expression of MjTyrRS and its loop-minimized mutants fused with the HA tag, the cell fragmentation supernatants and precipitates were carried out for SDS-PAGE, followed by the Western Blot test. The lanes on the cellulose-nitrate membrane are: M: partial bands of the 180 kDa Prestained Protein Marker; Lane 1: MjTyrRS precipitate fraction; Lane 2: MjTyrRS supernatant fraction; Lane 3: MjTyrRS Loop1 mutant precipitate fraction; Lane 4: MjTyrRS Loop1 mutant supernatant fraction; Lane 5: MjTyrRS Loop2 mutant precipitate fraction; Lane 6: MjTyrRS Loop2 mutant supernatant fraction; Lane 7: MjTyrRS Loop3 mutant precipitate fraction; Lane 8: MjTyrRS Loop3 mutant supernatant fraction.

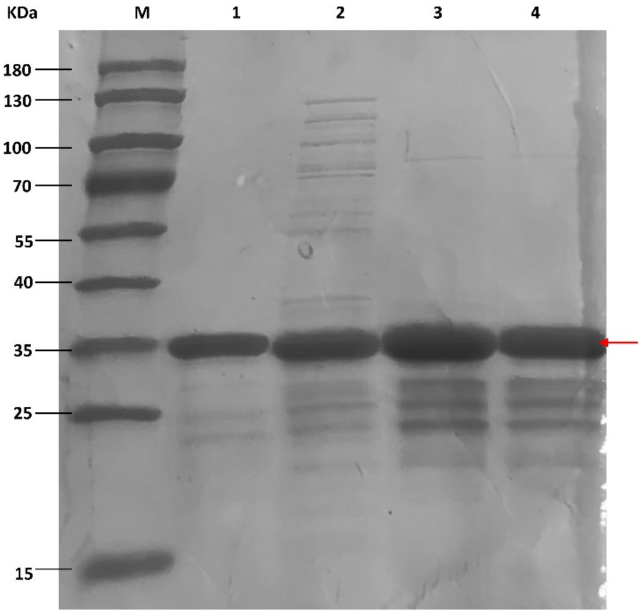

Figure S7. The SDS-PAGE result of purification of the MjTyrRS and its loop-minimized mutants by Co^{2+} affinity chromatography. The lanes in the gel are: M: 180 kDa Prestained

Protein Marker r; Lane 1: wild-type MjTyrRS; Lane 2: MjTyrRS Loop1; Lane 3: MjTyrRS Loop2; Lane 4: MjTyrRS Loop3. The red arrow points to the position corresponding to the size of MjTyrRS.

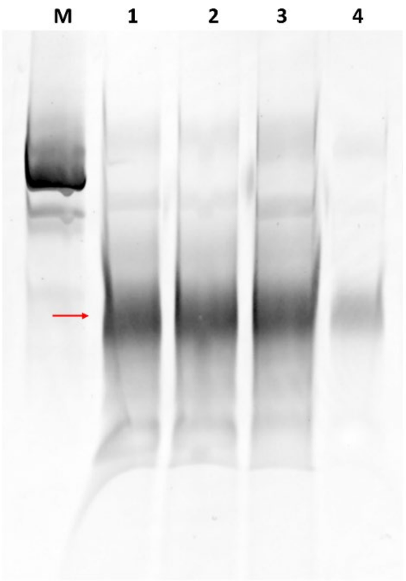

Figure S8. The denaturing polyacrylamide–urea gels result of the purified MjtRNA. The MjRNAs were resolved by electrophoresis on 7 M urea/12% acrylamide gels. The bands on the gel are: M: DNA template used for transcription as marker(139bp) 1~2: purified MjtRNA$_{CUA}$(77nt) 3~4: purified MjtRNA$_{UCUA}$(78nt). The red arrow indicates the position of the corresponding band of mjtRNA.

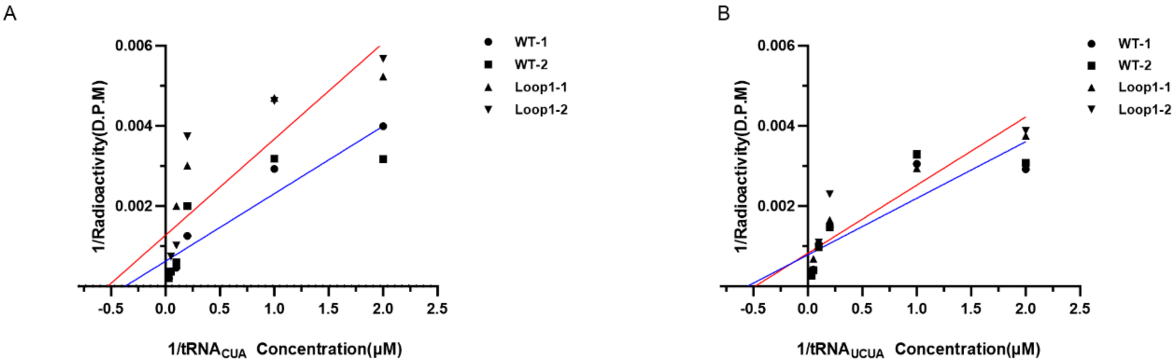

Figure S9. The aminoacylation assays of wild-type MjTyrRS and MjTyrRS Loop1 in response to (A) MjtRNA$_{CUA}$ and (B) MjtRNA$_{UCUA}$. The assay method was described in the **Aminoacylation assays in vitro**. The substrates for the reactions were triplet MjtRNA$_{CUA}$ and quadruplet MjtRNA$_{UCUA}$, respectively. The horizontal coordinates in the graph above represent the reciprocal of the substrate concentration, and the vertical coordinates represent the reciprocal of the measured radioactivity. The fitting curves of wild-type MjTyrRS and loop1 are shown in blue and red, respectively. Each set of experiments was repeated twice, distinguished by 1 and 2 after the group name.

Table S3. Kinetic parameter for tyrosylation by wild-type MjTyrRS and MjTyrRS Loop1.

	K_m(μM) for wt-MjTyrRS	K_m(μM) for MjTyrRS Loop-1
$MjtRNA_{CUA}$	4.74	1.88
$MjtRNA_{UCUA}$	1.77	1.82

tRNA preparation:

The MjtRNA used in this work, whose corresponding DNA sequences begin with an unfavorable nucleotide for transcription by T7 polymerase, was prepared by the 'transzyme' method. After transzyme transcription, the reaction was incubated at 60 °C for autocatalytic cleavage to produce the desired tRNA. The transcripts were then purified using a DEAE sepharose column, and the purified MjtRNA was obtained by elution using a buffer containing NaCl. The tRNA was then precipitated overnight by adding double-volume isopropanol. The precipitated MjtRNA was washed twice with ice-ethanol the following day and then dissolved in aminoacylation assay buffer.

Aminoacylation assays in vitro:

The MjtRNA transcripts were obtained by in vitro transcription, and the purified MjTyrRS and its loop-minimized mutants were obtained by affinity chromatography methods. Before the aminoacylation assay, the MjtRNA transcripts were renatured by heating for 2 minutes at 80 °C, followed by slow cooling to room temperature. Tyrosylation of RNAs was performed in 150 mM Hepes/KOH pH 7.5, 15 mM $MgCl_2$, 10 mM ATP,.5 mg/mL BSA, and 20 M dithioerythritol. ^{14}C isotope-labeled tyrosine was diluted to a radioactivity of 18.75 μci/mL. Kinetic parameters were determined in the presence of 2μM of MjTyrRS or its loop-minimized mutants and transcripts concentrations varying from 0.5μM to 30μM. After incubation at 37 °C for 5 min, aliquots were removed, spotted on Whatman 3mm paper, and precipitated by trichloroacetic acid. The incorporation of radioactive tyrosine was measured by liquid scintillation. Radioactivity values were obtained by a Hitachi Aloka LSC-8000. The kinetic constants K_m were derived from Lineweaver–Burk plots, and the data shown represent an average of at least two independent experiments.
